# Metal–Organic Frameworks Based on a Janus-Head Biquinoline Ligand as Catalysts in the Transformation of Carbonyl Compounds into Cyanohydrins and Alcohols

**DOI:** 10.1021/acs.cgd.2c00985

**Published:** 2022-11-08

**Authors:** Juana M. Pérez, Samuel Morales-Cámara, Francisco M. García-Salas, Noelia Ruiz-Cuevas, Mireya E. López-Vargas, Duane Choquesillo-Lazarte, Javier Cepeda, Jose A. García, Víctor Karim Abdelkader-Fernández, Antonio Rodríguez-Diéguez, Sara Rojas, Ignacio Fernández

**Affiliations:** † Dept. of Chemistry and Physics. Research Centre CIAIMBITAL, University of Almería, Ctra. Sacramento S/n, Almería 04120, Spain; ‡ Dept. of Inorganic Chemistry, University of Granada, Av. Fuentenueva S/n, Granada 18071, Spain; § Lab. de Estudios Cristalográficos. IACT, CSIC-UGR, Av. Las Palmeras N°4, Granada 18100, Spain; ∥ Dept. de Química Aplicada, Universidad del País Vasco (UPV/EHU). Paseo Manuel de Lardizabal, N° 3, Donostia-San Sebastián 20018, Spain; ⊥ Dept. de Física, Facultad de Ciencia y Tecnología, Universidad del País Vasco (UPV/EHU), Barrio Sarriena S/n, Leioa 48940, Spain

## Abstract

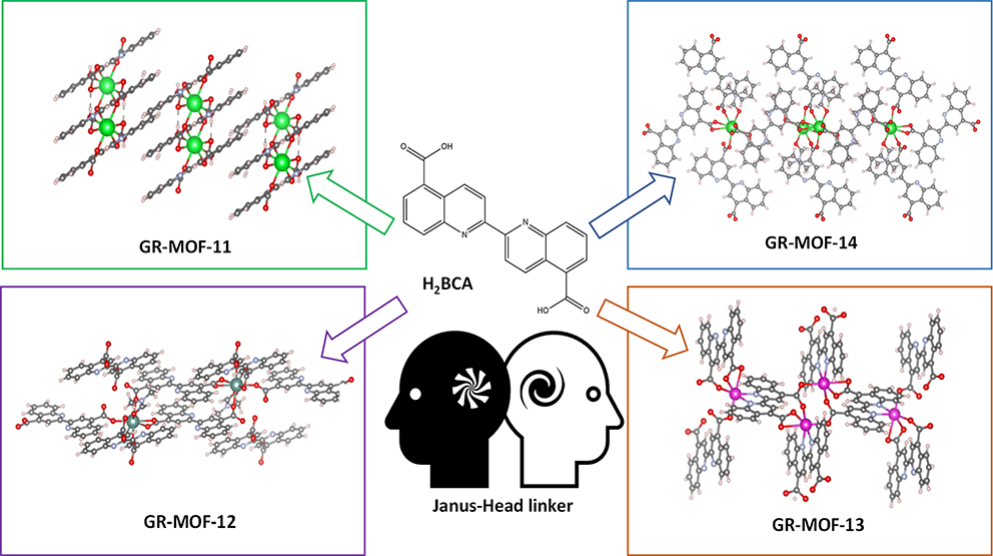

A new family of metal–organic frameworks (MOFs) named **GR-MOFs** with the chemical formula {[M_*x*_(BCA)_*y*_](H_2_O)_*z*_(DMF)_*w*_} (*x*,*y*,*z*,*w*: 1,1,2,0; 1,1.5,0,1; 1,2,2,1; and 1,1,0,2 for **GR-MOF-11** to **14**, respectively) based on s-block [M: Sr (**GR-MOF-11**), Ba (**GR-MOF-14**)] and d-block [M: Y (**GR-MOF-12**) and Cd (**GR-MOF-13**)] metals together with the biquinoline ligand 2,2′-bicinchoninic acid (H_2_BCA) has been synthetized by a solvothermal route and fully characterized by elemental and thermogravimetric analysis, Fourier transform infrared spectroscopy, photoluminescence, particle size distribution through optical microscopy, electrophoretic mobility, and finally, X-ray single-crystal and powder diffraction. The structural characterization reveals that these 2D and 3D MOFs possess a rich variety of coordination modes that maintained the Janus-head topology on the ligand in most of the cases. The new MOFs were studied in the catalyzed cyanosilylation and hydroboration of an extensive group of aldehydes and ketones, wherein the s-block metal-based MOFs **GR-MOF-11** and **GR-MOF-14** provided the highest efficiency ever reported in the MOF-catalyzed cyanosilylation of carbonyl compounds by using only 0.5 mol % of catalyst loading, room temperature, and solvent-free conditions. Furthermore, the hydroboration of ketones has been reported for the first time with this type of s-block metal catalysts obtaining from moderate to good conversions.

## Abstract 2

A new family of metal−organic frameworks (MOFs) named GR-MOFs based on s-block (Sr, Ba) and d-block (Y and Cd) metals together with the Janus-head ligand 2,2′-bicinchoninic acid have been synthetized and fully characterized. According with the previous use of this linker in detection and catalysis, the benefits of its integration in MOFs are reported here. The results presented shed some light on the potential of s-block metal-based MOFs as heterogenous catalysts.

## Introduction

During the last decade, the interest of the scientific community in the use of metal–organic frameworks (MOFs) has notably increased, mainly due to the precise functional ability to optimize their properties. MOFs are potentially porous crystalline materials based on a regular array of metal ions connected through organic linkers.^1^ Their potential applications in gas storage, gas and drug delivery, luminescence, environmental remediation, as well as their use as heterogeneous catalysts in organic transformations, among others, make them very valuable materials.^2,3^ Particularly, MOFs based on s-block metals^4−6^ have been less studied in comparation with the rest of the metals due to the ionic nature of the bonding interaction with carboxylate oxygen of the ligands normally used and the highly different electronegativity, which provides little room for the prediction and control over coordination geometry. Regardless of these challenges, s-block metal-based MOFs present several interesting features associated with these metals: (i) a strong M–OOC– interaction as a result of their high charge-density and ionic nature, (ii) their non-toxic nature or even essential in many biological processes, and (iii) their abundance in the earth crust, so they are considered cheap.^7,8^

In an attempt to obtain novel MOF materials, we have selected bicinchoninic acid or 2,2′-biquinoline 4,4'-dicarboxylic acid (H_2_BCA) as the Janus-head linker. The non-hindered character of the H_2_BCA molecule along the C_ipso_–C_ipso_ bond would *a priori* facilitate the coordination and formation of novel MOFs. H_2_BCA is normally used in the detection of different species in water (*i.e.,* proteins^9^ or organic molecules)^10^ but also as a homogeneous co-catalyst [*i.e.,* with Pd(PhCN)_2_Cl_2_ in the reductive amination of aldehydes^11^ or with CuCl_2_ in the oxidation of alcohols].^12^ Accordingly, it would be beneficial to integrate the H_2_BCA linker in an MOF to be used in the detection of interesting molecules or as heterogeneous catalyst in organic reactions. Among the most economically interesting reactions, the cyanosilylation of aldehydes and ketones toward the synthesis of cyanohydrins appears as a challenging process.^13^ This particular reaction is considered a benchmark transformation in the application of MOFs as heterogeneous catalysts.^14^ Among the previous MOFs reported in the cyanosilylation of aldehydes and ketones, their efficiency is relatively limited with less reactive ketones as substrates,^15−17^ with only one example of an s-block metal-based MOF.^18^

Herein, a new family of MOFs (named **GR-MOFs**) based on H_2_BCA as linker, and different s-block (Sr and Ba) and d-block (Y and Cd) metals is reported. Then, their high structural and colloidal stability is demonstrated, making them excellent candidates for sensing and heterogeneous catalysis. Particularly, we have studied their photoluminescent properties and their application and reuse in the transformation of carbonylic compounds into cyanohydrins and secondary alcohols, both of great interest to the fine chemicals industry.

## Experimental Section

### Synthesis of **GR-MOF-11** ([Sr(BCA)_2_]·2H_2_O)

0.03 mmol of 2,2′-bicinchoninic acid (H_2_BCA, 10 mg) was dissolved in 2 mL of *N,N*-dimethylformamide (DMF) and 1 mL of distilled water with heating at 90 °C. Separately, 0.03 mmol of Sr(NO_3_)_2_ (6 mg) was dissolved in 1 mL of distilled water. This last solution was added to H_2_BCA solution dropwise under stirring. The cloudy suspension obtained was placed in a closed vessel and heated to 95 °C for 24 h. X-ray quality crystals of **GR-MOF-11** were obtained during the heating process under autogenous pressure. Yield: 87%. Calcd Anal. for [SrC_20_H_10_N_2_O_4_]·2H_2_O: C, 51.55; H, 3.03; N, 6.01. Found: C, 51.09; H, 3.32; N, 6.17. Thermogravimetric analysis (TGA) calculated residue (SrO): 12.84%, obtained 22.43%.

### Synthesis of **GR-MOF-12** ([Y_2_(BCA)_3_] 2DMF)

0.03 mmol of H_2_BCA (10 mg) was dissolved in 2 mL of DMF and 1 mL of water with heating at 90 °C. In another glass vessel, 0.04 mmol of YCl_3_ (13 mg) was solved in 1 mL of water. This last solution was added to H_2_BCA solution dropwise under stirring. A cloudy suspension obtained was placed in a closed glass vessel and heated at 95 °C for 24 h. X-ray quality crystals of **GR-MOF-12** were obtained during the heating process under autogenous pressure. Yield: 57%. Calcd Anal. for [Y_2_C_30_H_15_N_3_O_6_]·2(C_3_H_7_NO): C, 51.63; H, 3.49; N, 8.36. Found: C, 57.40; H, 3.56; N, 8.56. TGA calculated residue (Y_2_O_3_): 16.72%, obtained 20.93%.

### Synthesis of **GR-MOF-13** ([Cd(BCA)]·DMF·2H_2_O)

0.03 mmol of H_2_BCA (10 mg) was dissolved in 3 mL of DMF with heating at 90 °C. In another glass vessel, 0.03 mmol of Cd(NO_3_)_2_ (9 mg) was dissolved in 1 mL of DMF. The metal solution was added to H_2_BCA dropwise under stirring. The resulting solution was placed in a closed glass vessel and heated at 95 °C for 24 h. After this time, X-ray quality crystals were obtained for **GR-MOF-13**. Yield: 82%. Calcd Anal. for [CdC_20_H_10_N_2_O_4_]·(C_3_H_7_NO) 2(H_2_O): C, 48.99; H, 3.75; N, 7.45. Found: C, 50.99; H, 2.95; N, 6.62. TGA calculated residue (CdO): 22.77%, obtained 26.50%.

### Synthesis of **GR-MOF-14** ([Ba(BCA)]·2DMF)

0.03 mmol of H_2_BCA (10 mg) was dissolved in 3 mL of DMF with heating at 90 °C. In another glass vessel, 0.03 mmol of Ba(NO_3_)_2_ (0,008 g) was dissolved in 1 mL of distilled water. This last solution was added to H_2_BCA dropwise under stirring. The resulting solution was placed in a closed glass vessel and heated at 95 °C for 24 h. After this time, X-ray quality crystals were obtained for **GR-MOF-14**. Yield: 76%. Calcd Anal. for [BaC_20_H_10_N_2_O_4_]·2(C_3_H_7_NO): C, 49.90; H, 3.87; N, 8.95. Found: C, 47.05; H, 3.88; N, 7.14. TGA calculated residue (BaO): 24.50%, obtained 37.69%.

Note here that although other alkali and alkaline-earth metals were studied, no new structures were obtained in the reaction with H_2_BCA as linker.

### General Procedure for the Cyanosilylation and Hydroboration Reactions

These processes were performed as previously described.^19^

### General Procedure for the Multi-Gram-Scale Catalytic Reaction

This procedure was performed similarly to those previously described.^19^ In a 3 mL vial equipped with a septum and a stirring bar, the catalyst was weight (**GR-MOF-11**, 11.64 mg, 0.5 mol %; or **GR-MOF-14**, 16.44 mg, 0.5 mol %). Then, the corresponding ketone **1f** or **1k** (5.0 mmol) followed by TMSCN (5.5 mmol, 1.1 equiv), and the reaction was stirred under an inert N_2_ atmosphere at room temperature for 24 h.

### Quantitative NMR Acquisition Parameters

^1^H NMR determinations of product conversions were carried out by comparing the aldehydic CHO signal in substrates **1**, or ketonic CH_3_ signal in substrates **3**, with those signals belonging from products **2** or **4**. The lack of signals coming from substrate **1** or **3** was assumed as conversions higher than 99%. The standard acquisition parameters were the same used in a previous report.^19^

## Results and Discussion

### Synthesis and Crystal Structure Description of GR-MOFs

**Figure 1:**
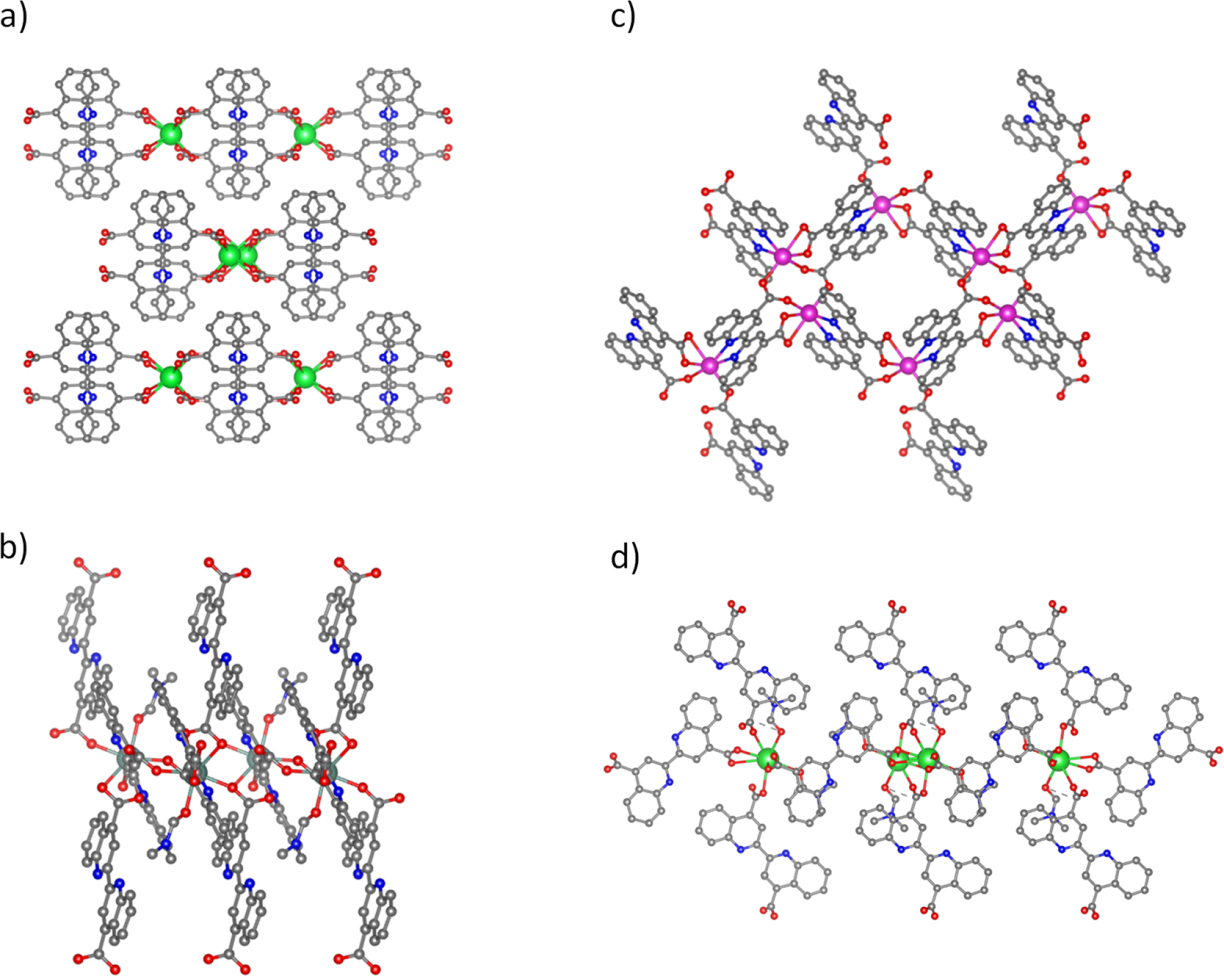
Perspective view of (a) **GR-MOF-11** (Sr), (b) **GR-MOF-12** (Y), (c) **GR-MOF-13** (Cd), and (d) **GR-MOF-14** (Ba).

**Scheme 1:**
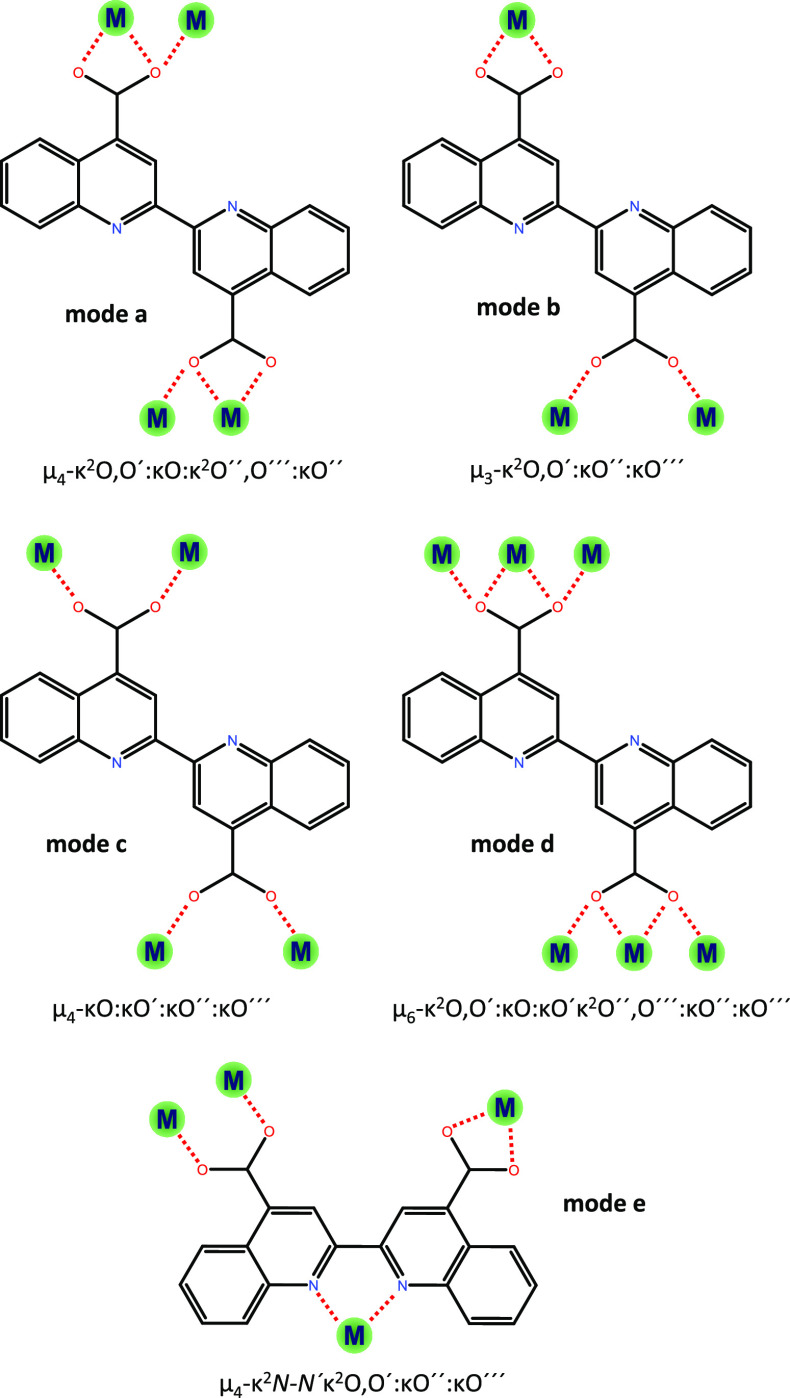
Coordination Modes Exhibited by BCA in the Construction of **GR-MOFs** Note that only the μ_4_-K^2^*N*–*N*K^2^O,O′:KO″:KO⁗mode does not retain the Janus-head topology of the ligand

A series of MOFs (named **GR-MOF-11** to **14**), based on the weak biquinolinic acid (H_2_BCA), were successfully prepared upon exhaustive optimization of reaction conditions. Briefly, the solvothermal reaction of H_2_BCA with strontium nitrate, yttrium(III) chloride, cadmium(II) nitrate, or barium nitrate (1:1 molar ratio) in a dimethylformamide (DMF)/water mixture or in pure DMF gives rise to the novel materials **GR-MOF-11** to **14**, respectively (see experimental section for further details). The resulting new MOFs with the formula [Sr(BCA)_2_]·2H_2_O (**GR-MOF-11**), [Y_2_(BCA)_3_]·2DMF (**GR-MOF-12**), [Cd(BCA)]·DMF·2H_2_O (**GR-MOF-13**), and [Ba(BCA)]·2DMF (**GR-MOF-14**) were prepared in high-purity single crystals suitable for their resolution by single-crystal X-ray diffraction (SCXRD, Figure 1). It should be noted here that, thanks to the different coordination modes exhibited by the ligand (Scheme 1), different multidimensional coordination polymers with different topologies have been obtained. Importantly, the Janus-head mode is retained in all of them except in **GR-MOF-13**, where the K^2^*N–N′* mode promotes the complete rotation of one of the quinolinic rings along the C_*ipso*_-C_ipso_ bond. As it is discussed below, this type of conformation is directly correlated to MOF catalytic activity. As revealed by SCXRD, **GR-MOF-11** crystallizes in the monoclinic space group *I*2/*a* with a 2D polymeric nature (Figure 1a). Here, the BCA ligand shows a μ_4_-K^2^O,Ó:KÓ:K^2^Ó́,Ó́́:KÓ́ coordination mode (Scheme 1) and each Sr is bonded to eight oxygen atoms, six from carboxylate groups of the linker and two water molecules. The Sr–O bond distances are in the range 2.506(2)–2.731(3) Å (see selected bond lengths (Å) and angles (°) of each compound in the Supporting Information, Section S3). The structure could be described as zig-zag chains of strontium atoms linked by the BCA, built thanks to stacking interactions between the aromatic rings of the linker and strong hydrogen bond interactions involving the O1 oxygen of the carboxylate group and the coordinated water molecule O1W (2.808 Å). On the other hand, **GR-MOF-12** crystallizes in the triclinic space group *P*1, with a 3D network (Figure 1b). The asymmetric unit is based on one yttrium atom, one and a half ligand, and one coordination of a DMF molecule. In this compound, the BCA ligand shows *a* and *b* coordination modes (Scheme 1). Yttrium atom possesses a YO_8_ coordination environment in which the Y–O bond distances are in the range 2.23(3)–2.48(3) Å, generating a Y–Y dinuclear unit in which the interaction is of a 3.919(9) Å distance. This nuclear unit connects two oxygens of the same carboxylic group of the linker, a single oxygen of the carboxylic group of BCA (two bonds of this type), and the carbonyl oxygen of DMF. With respect to the ligands, the planes containing the carboxylate groups coordinated to metals are not coplanar with the aromatic skeleton of the ligand, forming angles with values between 26.71 and 66.16°. Further, **GR-MOF-13** crystallizes in the monoclinic space group *P*2_1_/*n*, generating a 3D-coordination polymer with crystallization solvent molecules in its channels along the *a* axis (Figure 1c). The asymmetric unit is composed of one cadmium, one BCA linker, one crystallization water molecule, and one disordered crystallization DMF molecule. In this case, the BCA ligand shows a μ_4_-K^2^*N–Ń*K^2^O,Ó:KÓ́:KÓ́́ coordination mode (Scheme 1). The metal shows a very distorted octahedral environment in which the Cd is connected to four linker molecules. Specifically, the Cd atom is bonded to two oxygens of the same carboxylate group of BCA, one oxygen of the carboxylic group of BCA, and two nitrogens in the heterocycle of BCA. There are no solvent molecules (whether DMF or H_2_O) bonded to the metal center. The Cd–O bond distances are in the range of 2.272(3)–2.296(4) Å, while the Cd–N distances have values of 2.324(4) and 2.358(4) Å. Interestingly, this MOF shows channels (*ca.* 7 Å, considering van der Waals radii) along the *a* axis in which the solvent molecules are lodged. These water molecules are located within the channels thanks to strong hydrogen bonding interactions involving the oxygen atom (O19) pertaining to a carboxylate group and the oxygen (O2) of the water molecule. These two hydrogen bond distances have values of 2.880 and 2.982 Å, respectively. Finally, **GR-MOF-14** crystallizes in the monoclinic space group *P*2/*c*, generating a compact (pore size *ca.* 1.5 Å, considering van der Waals radii) 3D-coordination polymer (Figure 1d). Here, the BCA ligand shows *c* and *d* coordination modes (Scheme 1). The asymmetric unit is composed of one barium atom, one ligand, and two DMF coordination molecules. The barium metal has a BaO_8_ coordination environment and is coordinated to two molecules of DMF molecules by the oxygen atom of the carbonyl group and three molecules of BCA by establishing bonds with the two oxygens of the carboxylic group. The Ba–O bond distances are in the range of 2.712(4)–2.959(3) Å, generating a Ba–Ba interaction of 4.0770(5) Å. Finally, the planes containing the carboxylate groups coordinated to metals are not coplanar with the aromatic skeleton of the ligand, forming angles with values of 54.84 and 70.71°.

### Physicochemical Characterization

When compared with the free H_2_BCA linker, Fourier transformed infrared (FTIR) spectra of **GR-MOFs** (Figures S2–S5) show the bond formation between H_2_BCA and metal ions, with a significant shift of the carbonyl (C–O) peak from a carboxylate moiety at 1698 cm^–1^ to 1660, 1641, 1669, and 1666 cm^–1^ for **GR-MOF-11**, **12**, **13**, and **14**, respectively. The broad peak present at 3200–2300 cm^–1^ attributed to the stretching vibration of O–H bonds on H_2_BCA which clearly shifted in the MOF samples indicates the coordination of water to **GR-MOFs**. Further, the chemical composition of **GR-MOFs** was confirmed by elemental and thermogravimetric analysis (see the Experimental Section for further details, Figure S6). Regarding the potential accessible porosity, it was estimated considering the volume occupied by the constitutional MOF atoms compared to the whole cell volume. From all the series, **GR-MOF-14** shows a great potential accessible porosity (*ca.* 65 vol.% free *per* unit cell, respectively), while a moderate value (*ca.* 23 vol % free *per* unit cell) is obtained for **GR-MOF-12** and **13** (note that van der Waals radii have been considered for the calculation). The purity and reproducibility of the obtained polycrystalline **GR-MOF** samples were confirmed by a Le Bail profile (Figure S1).

### Photoluminescence Properties

As mentioned during the introduction, the presence of the BCA ligand possessing delocalized π-electrons gives these MOFs the opportunity of showing interesting photoluminescence (PL) properties. Thus, a complete PL characterization of the solid samples was conducted at variable temperatures.

**Figure 2:**
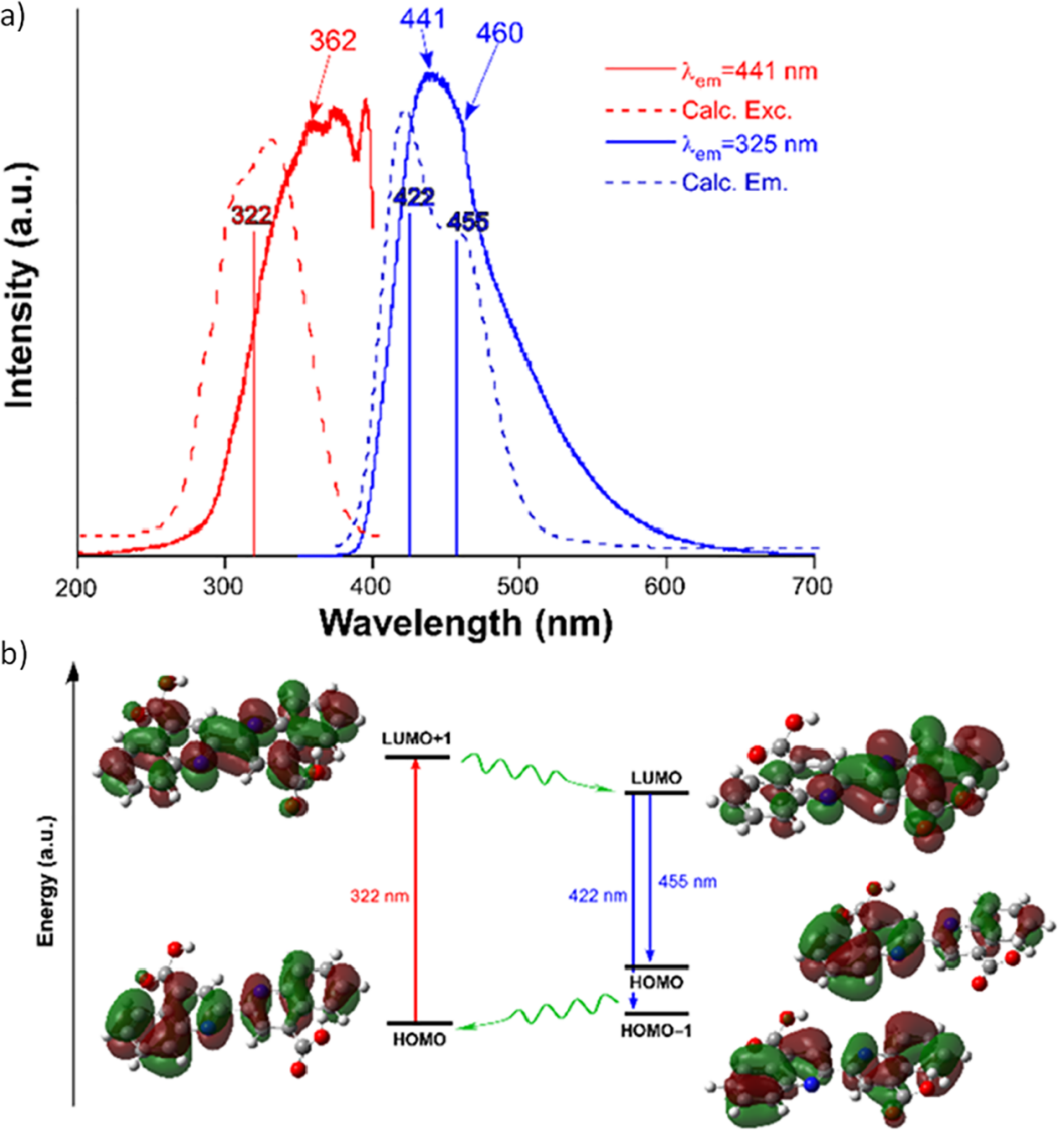
a) Experimental and TD-DFT computed excitation (red) and emission (blue) spectra of the H_2_BCA ligand. The most remarkable computed excitation lines are also shown. (b) Scheme of the energy distribution of the molecular orbitals involved in the main excitations.

At room temperature, the solid sample of H_2_BCA shows a wide band with the maximum centered at λ_em_ = 441 nm and a slightly perceptible shoulder peaking at λ_em_ = 460 nm when excited under a 325 nm laser light (Figure 2a). Moreover, the curve possesses a long slowly descending tail up to 600 nm. Focusing on the emission maxima, the excitation spectrum reveals a main rather structured band with three maxima, the first one sited at 362 nm. It is therefore reasonable to think that the excitation used to collect the emission spectrum falls within the main band. In order to get a deeper insight into the PL scenario of this ligand, time-dependent density functional theory (TD-DFT) calculations were also performed on the molecule (model 1, see computational details). As observed in Figure 2a, both the calculated excitation and emission spectra reproduce well the experimental ones, especially considering the shape of their bands, observing a good overlap for the emission spectra but a larger red shift of *ca.* 45 nm for the excitation spectra. Among all the computed excitation lines, the most remarkable excitation is sited on 322 nm, so it may be taken as a reference for the experimental excitation. This energy gap fits with the HOMO → LUMO + 1 transition that involves a reorganization of the π clouds of the ligand, with the HOMO being a bonding orbital and the LUMO + 1 an antibonding one (Figure 2b). After the excitation, the optimization of model 1 to the lowest-lying geometry reveals that the emission proceeds from the excited LUMO following two equivalent paths: (i) the corresponding maxima band (at 422 nm) related with the HOMO ← LUMO transition and (ii) that reproducing the shoulder (at 455 nm) described by the HOMO–1 ← LUMO transition. It is worth noticing that these two emissions involve molecular orbitals of the same nature as those previously described during the excitation. In line with these calculated singlet-to-singlet transitions, the measurement of the decay curve for the H_2_BCA linker confirmed the fluorescent nature of the emission, giving a short lifetime of 2550(22) ps (Figure S7). To finish with the ligand characterization, the emission was also measured at a low temperature, demonstrating its stability but with the expected intensity increase due to the freezing of the vibrations of the molecule (see Figure S8).^20,21^

**Figure 3:**
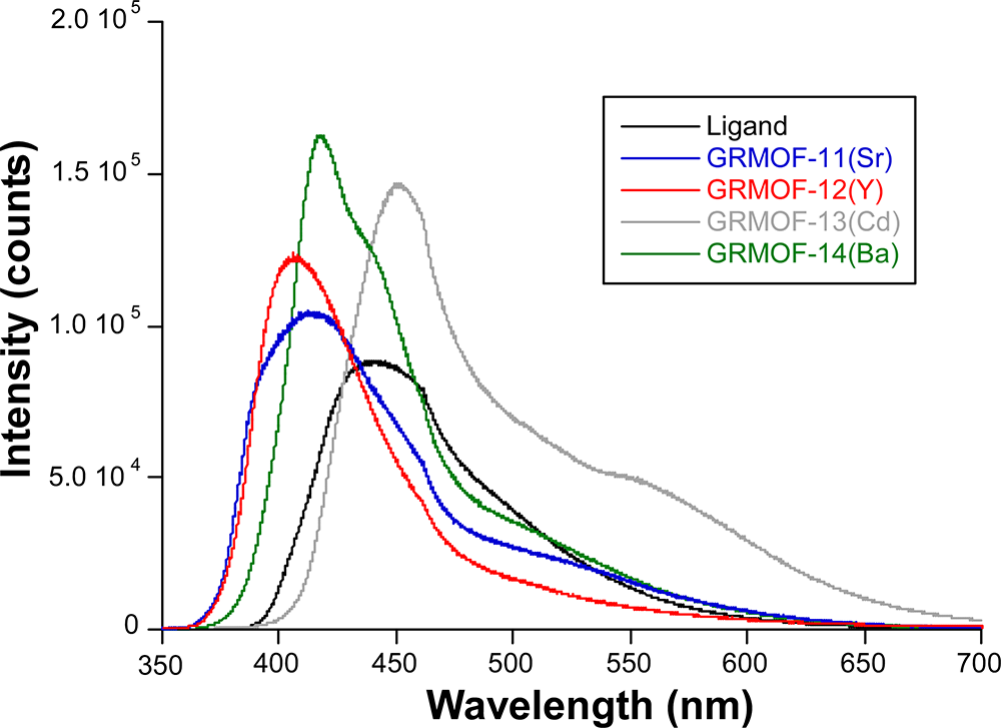
Emission spectra of the H_2_BCA ligand and **GR-MOFs** recorded under the same experimental conditions.

Once the biquinolinic ligand is coordinated to closed-shell metal ions (with no characteristic metal-centered emission), a stronger intensity is observed compared to the free ligand as many vibrational modes are restricted (Figure 3). However, the ligand-centered emission experiments demonstrated slight or significant changes depending on the conformations acquired by the organic molecule in the crystal structure because of its coordination.

In essence, **GR-MOF-11** and **12** present a very similar emission pattern to that of the ligand although the maximum of the main band is substantially blue-shifted (from 441 to 405 nm). Remarkably, both spectra reproduce the subtle shoulder found in the ligand’s spectrum at λ_em_ = 460 nm, a fact that makes one conclude that the LUMO–HOMO energy gap is maintained in these compounds. On its part, the barium-based **GR-MOF-14** presents an intermediate blue-shifting with a maximum of the main band at λ_em_ = 418 nm and a well-defined shoulder coincident with the ligand’s band maximum (λ_em_ = 443 nm). Further, despite the changes in the emission profile, it can be confirmed that it is a ligand-centered luminescence given the lifetimes (3669(69), 2048(22), and 2588(25) ps for **GR-MOF-11**, **12**, and **13**, respectively) estimated from the decay curves which are very similar to those of the free H_2_BCA ligand (2550(22) ps). On the contrary, the cadmium-based **GR-MOF-13** presents, perhaps, the most different emission spectrum as it shows a main wide band centered at λ_em_ = 455 nm in addition to a new band peaking at *ca.* 550 nm that is overlapped with the tail of the latter. In this case, although the fluorescent emission (λ_em_ = 455 nm) remains similar to that of the ligand, the emission at λ_em_ = 550 nm is much long-lived and possesses a lifetime of 52(8) ms, a value that makes it be considered within the range of long-lived phosphorescence.^22^ The origin of this phosphorescence could not be further studied but related to the different conformation acquired by the ligand in this compound since it is unique, showing a chelating ring with the aromatic nitrogen donor atoms.

To conclude with the PL characterization, the variable-temperature emission was also studied. The expected enhancement of fluorescent intensity was observed, with no further significant changes, except for **GR-MOF-13**, which exhibits a peculiar behavior. In the **GR-MOF-13** spectra, the band peaking at 550 nm is not enhanced as previously observed for other CPs based on Cd(II)^23−26^ and remains below the strongest band at 455 nm in such a way that a dominant blue emission is observed at a low temperature.

### Chemical Stability and Electrophoretic Behavior

The potential of MOFs as catalysts might be hampered by their poor stability under the working conditions. The thermal stability of **GR-MOFs** was therefore studied by TGA (Figure S6). In all of them, a first slight weight loss from room temperature to *ca.* 130 °C is attributed to the elimination of water adsorbed on the external surface of the crystal (particularly visible in **GR-MOF-12** and **13**). The following weight loss, between 130 and 275–400 °C, might correspond to the removal of water and DMF molecules. The following progressive mass loss starting at 450 °C corresponds to the decomposition of the organic ligand. The accurate quantification of the obtained thermal residue according to the proposed formula is also included in the experimental section. It should be noted that in the cyanosilylation reaction studied herein, no additional heating is necessary. However, we believe that it is important to check the thermal stability of these materials considering their potential use in other significant catalytic reactions.

**Figure 4:**
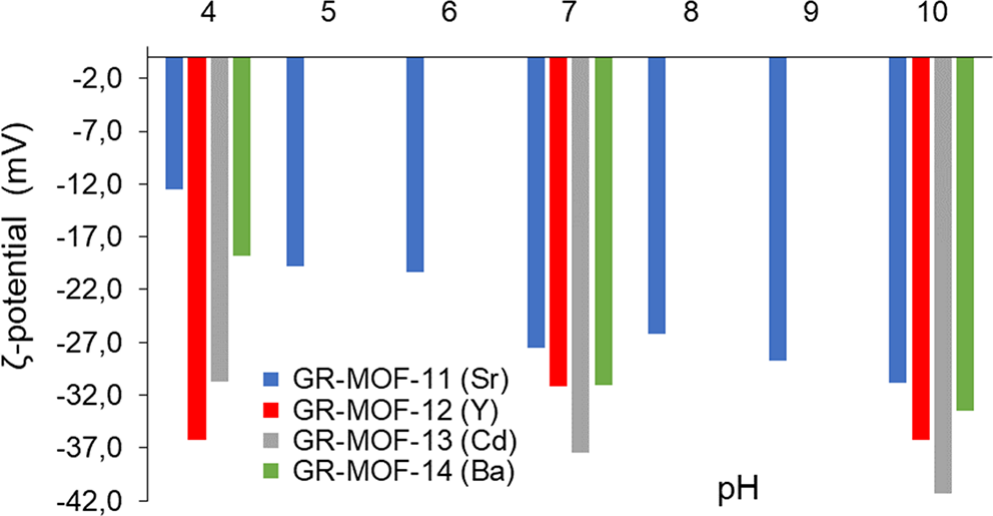
Comparison of the *ζ*-potential as a function of pH for **GR-MOF-11** to **14**. All the measurements were performed with constant conductivity of 330 μS/cm ([NaCl] = 3.7 mM).

Further, the chemical stability and reactivity of a catalyst is determined by its surface chemistry. Here, the surface charge and colloidal stability of **GR-MOF** materials were assessed by electrophoretic mobility measurements (Figure 4). Surface charge variations as a function of pH are observed for all the assayed MOFs at a fixed conductivity of 330 μS/cm. Negative values of the *ζ*-potential for all the **GR-MOFs** suggest that dissociation of non-bonded acidic groups have occurred, and the lower the magnitude the more stable and the less the tendency to aggregate. In addition, the *ζ*-potential variation associated to the pH change from 4 to 10 is higher in **GR-MOF-11** and **14** (from −12.5 ± 0.8 to −30.8 ± 1.1 and from −18.8 ± 0.9 to −33.5 ± 0.9, respectively) suggesting a higher number of available carboxylic acids for deprotonation and a higher number (or more accessible) of coordination sites for proton adsorption or interaction in these MOFs. Among all, **GR-MOF-13** shows a more negative *ζ*-potential value in the entire pH range (up to −41.3 ± 0.5 mV at pH 10), associated with a major colloidal stability (Table S6).

Particle size distribution of the four catalysts was also examined using optical microscopy (Figures S13–S16). Solutions of the four catalysts were prepared (2.5% w/w), and the deposited fraction of each catalyst was approximately 82, 70, 53, and 55% for **GR-MOF-11** to **14**, respectively. The supernatants were analyzed by mobility measurements, whereas the deposited pellets were studied by optical microscopy. Crystalline needles of 65 ± 33 μm in the case of **GR-MOF-11** and amorphous particles with less than 10 μm for **GR-MOF-12** and **13** and of about 21 ± 15 μm for **GR-MOF-14** were observed.

### Catalytic Activity

**Scheme 2:**
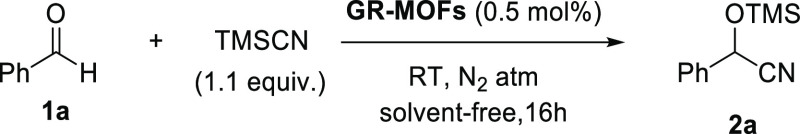
Benchmark Reaction Used in the Catalytic Performance of the Different **GR-MOFs**

In order to compare the catalytic activity of the whole set of **GR-MOFs** with those previously reported,^19^ the cyanosilylation reaction of benzaldehyde (**1a**) with TMSCN was tested using 0.5 mol % of the catalyst, and the benchmark reaction conditions were generally assayed (Scheme 2). As it is discussed further below, the experimental times required for quantitative conversions did not recommend lower loading of the catalyst, so the study was limited to 0.5 mol % or higher. In any case, this reaction provides an efficient route for the selective synthesis of cyanohydrins, which are key derivatives in the synthesis of compounds with chemical and pharmaceutical industrial applications.^27^

Initially, the conversion of the reactions after 7 h at room temperature were analyzed using ^1^H NMR spectroscopy, obtaining full conversion to the desired product with **GR-MOF-11** and **14**, and moderate to good conversions with **GR-MOF-12** and **13**. Interestingly, those s-block metal-based MOFs (Sr and Ba, **GR-MOF-11** and **14**, respectively) provide the highest conversion (99% for both), whereas when using those based on Y and Cd (**GR-MOF-12** and **13**), the conversion was remarkably reduced (71 and 46%, respectively). However, the conversion for **GR-MOF-12** increased up to 96% with 16 h of reaction time (entry 1, Table 1), whereas for **GR-MOF-13**, it remained modest (62%). Recall that the latter was the MOF in which the ligand does not maintain the Janus-head conformation within the final architecture. The latter statement points out to a direct correlation between the plausible Janus-head ligand conformation within a positive catalytic activity.

**Table 1:**
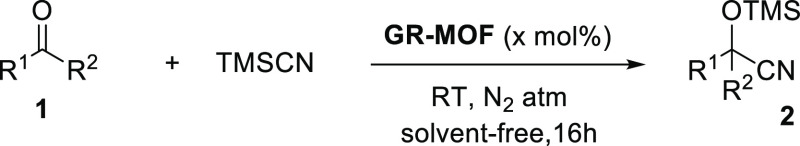
Results of Cyanosilylation of Aldehydes and Ketones in the Presence of Different Catalysts **GR-MOF** at Room Temperature^a^

entry	*R*^1^	*R*^2^	no.	GR-MOF-11 (0.5 mol %)^b^	GR-MOF-12 (1 mol %)^b^	GR-MOF-13 (0.5 mol %)^b^	GR-MOF-14 (0.5 mol %)^b^^]^
1	Ph	H	**2a**	>99	96	62	>99
2	4-MeOC_6_H_4_	H	**2b**	>99	96	38	>99
3	4-ClC_6_H_4_	H	**2c**	>99	>99	29	>99
4	2-Pyridine	H	**2d**	>99	>99	>99	>99
5	Et	H	**2e**	>99	>99	>99	>99
6	Ph	Me	**2f**	78 (92)^c^	71	0	83 (98)^c^
7	4-MeOC_6_H_4_	Me	**2g**	79	31	0	84
8	4-ClC_6_H_4_	Me	**2h**	93	43	0	89
9	2-Pyridine	Me	**2i**	>99	>99	75	>99
10	Et	Me	**2j**	>99	>99	30	>99

a Reaction carried out using compounds **1** (0.25 mmol) and TMSCN (40 μL, 0.275 mmol, 1.1 equiv.) at room temperature under an inert nitrogen atmosphere.

b Conversions (relative to compound **1**) determined by ^1^H NMR of the reaction crude.

c Conversion (relative to ketone **1**) determined by ^1^H NMR of the reaction crude after 24 h of reaction using a gram-scale reaction (5 mmol of **1f**). Note that only 8% of conversion was obtained after 14 h when the blank reaction was conducted.

**Figure 5:**
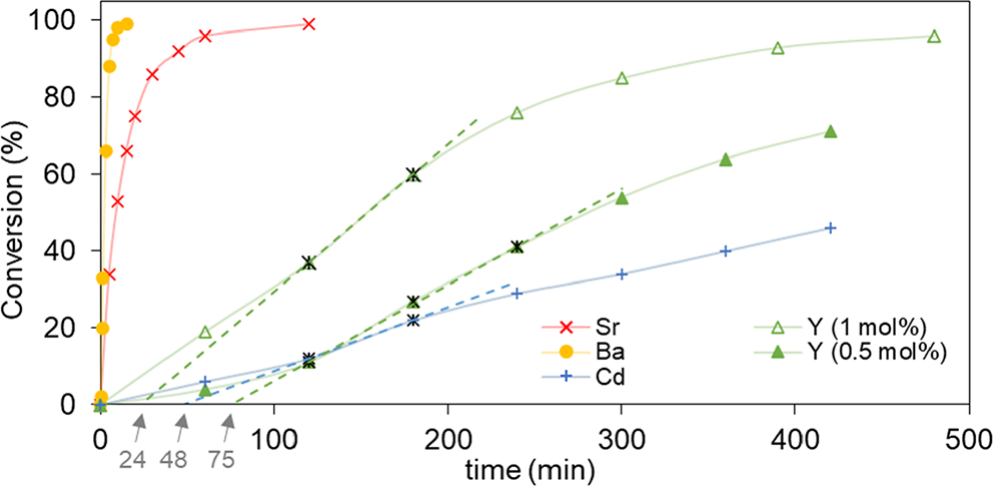
Kinetic profile of the studied reaction using **GR-MOF-11** [Sr, red(x)], **GR-MOF-12** [Y, green(▲)], **GR-MOF-13** [Cd, blue(+)], and **GR-MOF-14** [Ba, orange(●)] with a ratio of **1a**/TMSCN of 1:1.1 under an inert atmosphere at room temperature.

When analyzing the kinetic profiles of the reactions (Figure 5), expected higher initial reaction rates were observed for **GR-MOF-11** and **14**, with less than 45 and 15 min to reach completeness, respectively. On the contrary, significantly slower processes were observed for **GR-MOF-12** and **13**, probably related with the required induction periods (48 and 75 min for **GR-MOF-12** and **13**, respectively). It should be noted that in an attempt to improve the data obtained with **GR-MOF-12**, the catalyst loading was increased up to 1 mol %, reducing the induction period to 24 min and the time to reach full conversion to 8 h.

Making use of the data obtained in the kinetic profiles, the turnover frequency (TOF) for all the **GR-MOFs** was calculated as a function of conversion (Figures S21-S24), obtaining a maximum TOF of 2640 h^–1^ at 1.5 min of reaction time (33% of conversion) with **GR-MOF-14** and a second top value of 816 h^–1^ at 5 min of reaction time (34% of conversion) for **GR-MOF-11**. These results remarkably overpass those found by our group with Y- and Eu-based MOF catalysts^19,48^ but also those ever reported with any MOF based on lanthanide, Cd, or Ba metals (maximum TOF reported, 384 h^–1^) (Tables S8 and S9, note that Sr-based MOFs have never been reported in these catalytic reactions).^18,28−34^

**Figure 6:**
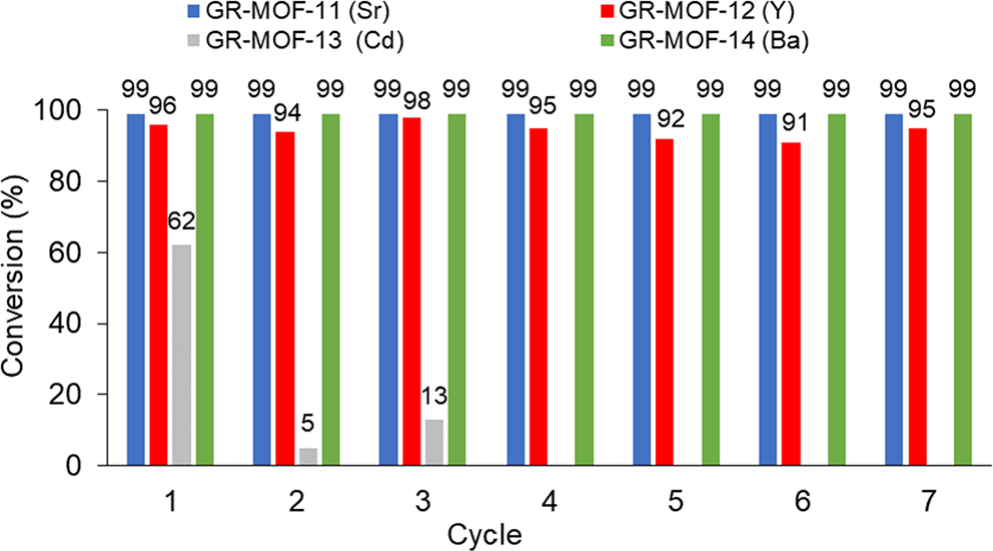
Recyclability of **GR-MOF** catalysts during seven consecutive cycles.

The potential recyclability of the catalysts was also considered as they can be easily isolated from the reaction solution by centrifugation. All the **GR-MOFs** could be recycled up to seven times without any erosion of catalytic activity, except for **GR-MOF-13**, which shows a decrease in the catalytic properties after the first cycle (Figure 6).

Regarding the chemical stability of **GR-MOF-11**, **12**, and **14**, inductively coupled plasma mass spectrometry (ICP–MS) analyses were performed before and after the reaction cycles, obtaining the same concentration of metal. These observations suggest that these three MOFs act as true heterogeneous catalysts.

Green chemistry metrics^35−38^ such as atomic economy (AE), mass intensity (MI), reaction mass efficiency (RME), and carbon efficiency (CE) were also calculated in order to evaluate if the overall transformation is eco-friendly and overcomes health and environmental problems derived from the chemical industry (Table S7). The obtained values of 99.9% of AE, 1.06 for MI, 95.4% for RME, and 96.5% for CE are comparable to those described previously for related MOFs.^19,37,38^

The general applicability of the reaction was investigated using a wide range of different aldehydes. The reaction with aromatic aldehydes bearing substituents of different natures in the *para*-position included electron-donating (OMe) and electron-withdrawing (Cl) groups providing excellent conversions when **GR-MOF-11**, **12**, and **14** were tested, but again, in the case of **GR-MOF-13**, moderate conversions were reached (entries 2-3, Table 1). These results agree with the stability results, and a likely degradation of the **GR-MOF-13** framework may take place. The use of heteroaromatic and aliphatic aldehydes led to the complete transformation to the corresponding silyl ether cyanohydrin when using all the catalysts (entries 4-5, Table 1), which could be explained in terms of coordination abilities in the former and due to small steric hindrance and easiness to get into the channels for the latter. As expected, the use of less reactive and sterically demanding ketones (entries 6–8, Table 1) gave good to excellent results with catalysts **GR-MOF-11** and **14** and again poor to moderate results with **GR-MOF-12** and **13**. Excellent conversions were obtained when using heteroaromatic or aliphatic ketones (entries 9-10, Table 1), except for **GR-MOF-13**, which yielded poor to moderate conversions. In most of the MOF-catalyzed reactions, the active sites are described to be Lewis acid metals, the activities of which are powered or lowered by the type of ligand, its coordination abilities, and the coordination mode of both the metal and ligand employed as building blocks. In fact, variables such as coordination number and reduced pore size could be playing an important role in helping the substrate to get into the channels of the MOF and therefore facilitating the reaction. In this sense, two possible catalytic reaction pathways have been proposed for this cyanosilylation process.^19^ In both of them, the coordination of the carbonylic substrate to the metal center is proposed as the first step of the reaction, favoring the subsequent nucleophilic attack of the TMSCN. Our results agree well with this hypothesis, and in the case of those based on s-block metals (**GR-MOF-11** and **14**), our proposed mechanism includes this coordination of the carbonyl step as the most critical. In the hydroboration reaction, previous studies have shown a first-order dependence on the MOF and ketone concentrations and a zeroth-order dependence on the HBpin concentration.^39^ The described mechanism involves a first step wherein HBPin reacts with the MOF to furnish [MOF]–H, then a second step that involves the insertion of the C=O into the already generated [MOF]–H, and then a third step wherein another molecule of HBPin furnishes the borate ester product and regenerated [MOF]–H that enters back in the cycle.

Finally, the two best MOF systems based on s-block metals (**GR-MOF-11** and **14**) were used in order to evaluate their performance at a higher scale. For that, we decided to synthesize 2-(2,4-difluorophenyl)-2-((trimethylsilyl)oxy)propanenitrile (**2k**) and 2-(phenyl)-2-((trimethylsilyl)oxy)propanenitrile (**2f**) in a multigram scale (Scheme 2) since they represent examples of useful precursors of the potent fungicide 5-(2,4-difluorophenyl)-4-thioxo-5-methyl-3-(phenylamino)oxazolidin-2-one^40^ or the active agrochemical 5-(phenyl)-4-imino-5-methyl-3-(phenylamino)oxazolidin-2-one.^41^ Full conversions to the desired product **2k** were obtained with isolated yields of 87% (1077 mg) and 97% (1238 mg) using catalysts **GR-MOF-11** and **14**, respectively. For the precursor **1f** (entry 6, Table 1), the isolated yields obtained were 90% (937 mg) and 95% (1041 mg) for catalysts **GR-MOF-11** and **14**, respectively.

**Scheme 3:**
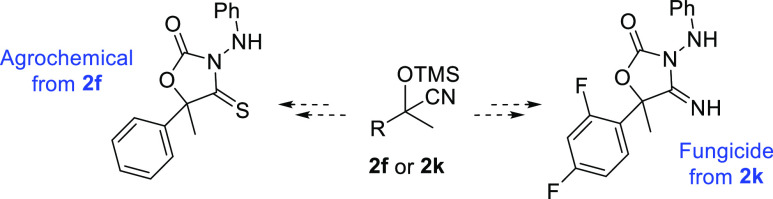
Application of the Cyanohydrin Products **2k** or **2f** toward the Synthesis of a Fungicidal as Agrochemical Active Compound

With these results in hand and encouraged by the good results obtained in the cyanosilylation reactions, we decided to test the four **GR-MOFs** in the less explored hydroboration reaction of ketones (Table 2). Catalytic hydroboration of carbonyl compounds is a straightforward methodology for the construction of boronic compounds that might be transformed into a great variety of organic functional groups.^42^ In fact, the reduction of this group represents one of the powerful tools for the synthesis of highly valuable alcohols that are present in numerous synthetic interesting targets.^43,44^ The use of an MOF as a catalyst in the hydroboration reaction has been slightly explored and only with those based on Mg,^39^ Ti,^45^ and Co^46,47^ and never with supramolecular entities built with ligands of potential Janus-head conformation such as BCA. Interestingly, the known reports required the use of a solvent such as hexane or toluene, which is a drawback from the green chemistry and economic viability points of view. Here, the reaction was carried out under solvent-free conditions with aromatic, heteroaromatic, and aliphatic ketones, obtaining similar results with all the catalysts (Scheme 3). Unfortunately, the use of *p*-substituted aromatic ketones whether bearing electron-donating or electron-withdrawing groups afforded low conversions (entries 2-3, Table 2). However, the conversion increased from *p*-chloro (**3c**) to *m*-chloro (**3d**) and *o*-chloroacetophenone (**3e**) (entries 3–5, Table 2). When the substrate of choice was 1-(pyridin-2-yl)ethan-1-one (**3f**), the reaction took place with very good to excellent conversions (entry 6, Table 2), whereas in the case of the aliphatic 2-butanone (**3g**), the results were from poor to moderate except for **GR-MOF-14**, which provided an excellent conversion of 94% (entry 7, Table 2).

**Table 2:**
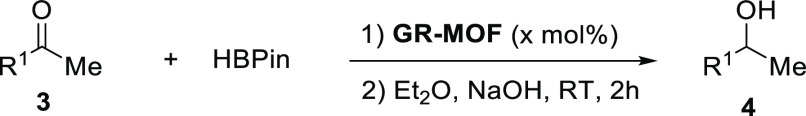
Results of Hydroboration of Ketones in the Presence of Different **GR-MOF** Catalysts at Room Temperature^a^

entry	*R*^1^	no.	GR-MOF-11 (0.5 mol %)^b^	GR-MOF-12 (1 mol %)^b^	GR-MOF-13 (0.5 mol %)^b^	GR-MOF-14 (0.5 mol %)^b^
1	Ph	**4a**	93	70	79	43
2	4-MeOC_6_H4	**4b**	0 (10)^c^	19 (46)^c^	13	4
3	4-ClC_6_H4	**4c**	0 (19)^c^	35 (48)^c^	0	49
4	3-ClC_6_H4	**4d**	82^d^	68^d^	31	35
5	2-ClC_6_H4	**4e**	84	89	63	75
6	2-Pyridine	**4f**	89	77	97	97
7	Et	**4g**	31 (78)^c^	69 (85)^c^	35	94

a Reaction carried out using compounds **3** (0.25 mmol) and HBPin (40 μL, 0.275 mmol, 1.1 equiv.) at room temperature under an inert nitrogen atmosphere.

b Conversions (relative to ketone **3**) determined by ^1^H NMR of the reaction crude.

c Conversion obtained after 4 days of reaction.

d Conversion obtained after 48 h of reaction.

## Conclusions

In summary, we have synthesized and characterized a new set of metal–organic systems based on Sr, Y, Cd, and Ba as metals and the biquinolinic ligand H_2_BCA. These new materials of the general formula {[M_x_(BCA)_y_](H_2_O)_z_(DMF)_w_} have been fully characterized by a plethora of techniques including X-ray single-crystal and powder diffraction and PL and electrophoretic measurements among some other methods such as optical microscopy, TGA, FTIR, and elemental analysis. Further, these supramolecular complexes have been employed in the solvent-free cyanosilylation and hydroboration of ketones and aldehydes, avoiding the use of volatile organic compounds as solvents and favoring the transformation to the final product under green conditions at room temperature and using reduced catalyst loadings of 0.5 to 1.0 mol %. Importantly, the barium catalyst **GR-MOF-14** in which the Janus-head ligand topology is retained showed an extraordinary activity in the cyanosilylation reaction with a TOF value of 2640 h^–1^ at 1.5 min of reaction time (33% of conversion), with the impressive possibility of recyclability for at least seven cycles. The application of more hindered Janus-head ligands in the construction of 2D and 3D MOFs and their use in tandem reactions involving the different metal centers are currently ongoing in our laboratory.

## Supporting Information Available

Crystallographic data, PXRD, FTIR, TGA, PL measurements, particle size distribution, electrophoretic mobility, TOF, green chemistry metrics, characterization of products, and recyclability studies (PDF)
