# Supplementary material for: Metal–Organic Frameworks Based on a Janus-Head Biquinoline Ligand as Catalysts in the Transformation of Carbonyl Compounds into Cyanohydrins and Alcohols
Source: Cryst Growth Des. 2022 Nov 8;22(12):7395–404. doi: 10.1021/acs.cgd.2c00985 (PMC9733562; doi:10.1021/acs.cgd.2c00985)
Supplement: Supplementary file 1 — cg2c00985_si_001.pdf [file cg2c00985_si_001.pdf]

Supporting Information

Metal-organic frameworks based on a Janus-head biquinoline ligand as catalysts in the transformation of carbonyl compounds into cyanohydrins and alcohols

Juana M. Pérez,[a,‡] Samuel Morales-Cámara,[b,‡] Francisco M. García-Salas,[a] Noelia Ruiz-Cuevas,[b] Mireya E. López-Vargas,[a] Duane Choquesillo-Lazarte,[c] Javier Cepeda,[d] Jose A. García,[e] Víctor Karim Abdelkader-Fernández,[b] Antonio Rodríguez-Diéguez,[b] Sara Rojas[*,b] and Ignacio Fernández[*,a]

[a] Department of Chemistry and Physics, Research Centre CIAIMBITAL, University of Almería, Ctra. Sacramento, s/n, 04120, Almería, Spain. Email: ifernan@ual.es

[b] Departamento de Química Inorgánica, Facultad de Ciencias, Universidad de Granada, Av. Fuentenueva s/n, 18071 Granada, Spain. Email: antonio5@ugr.es

[c] Laboratorio de Estudios Cristalográficos, IACT, CSIC-UGR, Av. Las Palmeras nº4, 18100 Granada, Spain.

[d] Departamento de Química Aplicada. Universidad del País Vasco (UPV(EHU). Paseo Manuel de Lardizabal, nº 3, 20018 Donostia-San Sebastián, Spain.

[e] Departamento de Física, Facultad de Ciencia y Tecnología, Universidad del País Vasco (UPV/EHU). Barrio Sarriena s/n, 48940 Leioa, Spain.

Table of Contents:

1 Materials and Characterization 3
2 Crystallographic data 5
3 Selected bond lengths and angles data 7
4 PXRD 11
5 FTIR characterization 12
6 Thermogravimetric analyses (TGA) 14
7 Photoluminescence measurements 15
8 Particle size distribution of catalysts 19
9 Study of ζ-potential at different pH values 24
10 TOF of GR-MOFs 27
11 Green chemistry metrics 29
12 Characterization Data of Products 30
13 Catalyst Recyclability 33
14 Results obtained with different lanthanides MOF catalysts 34
15 References 38

1 Materials and Characterization

. All experiments involving moisture-sensitive compounds were performed under an inert atmosphere of N_2 using standard techniques. Unless otherwise indicated, reagents and substrates were purchased from commercial sources and used as received. Solvents not required to be dry were purchased as technical grade and used as received. Conversion values relative to the limiting reagent were calculated from the ^{1}H NMR spectra of the reaction crudes. Isolated products were obtained after centrifugation (8000 rpm, 3 min) and washed with dichloromethane (2 x 0.5 mL) in order to remove the catalyst or column chromatography in silica gel using hexane as eluent. Infrared spectroscopy (IR, attenuated total reflection-ATR) measurements of **GR-MOF** catalysts were recorded in a FT-IR Bruker Alpha spectrophotometer, whereas IR spectra of H_2BCA was recorded on a Nicolet 6700 FTIR spectrophotometer (Thermo Phisher Scientific, TX, USA) with samples as KBr disks. Thermogravimetric analysis (TG/DTA) were performed on a TG-Q500 TA Instruments thermal analyser from room temperature to 800 ˚C under a synthetic air atmosphere (79% N_2/21% O_2) at a heating rate of 10 ˚C min^{-1}. NMR spectra were measured in a Bruker Avance III 300 spectrometer equipped with a direct double SmartProbe BBFO ^{1}H/BB(^{19}F) probe. Chemical shifts are reported in parts per million

(ppm) relative to TMS as spectral reference. Photoluminescence spectra were recorded on an Edinburgh Instruments FLS920 spectrometer equipped with a closed cycle helium cryostat. All measurements were performed under high vacuum (of *ca.* 10−9 mbar) to avoid the presence of oxygen or water in the sample holder. For steady-state measurements, an IK3552R-G HeCd continuous laser (325 nm) was used as the excitation source, whereas a Müller-Elektronik-Optik SVX1450 Xe lamp was employed to collect the excitation spectra. Luminescence collected in the UV-Vis region was analyzed with a photomultiplier tube (PMT) coupled to the spectrometer. On the other hand, the decay curves were measured using a μF900 microsecond pulsed lamp or a pulsed laser diode LDH-P-C-375 (λ = 375 nm) depending on the emission longevity. Electrophoretic mobility and ζ-potential measurements were made in a ZetaSizer Nano-Z ZEN2600 instrument, DTS1070 cell type, and laser wavelength of 632.8 nm. Samples were prepared at 0.05% w/w, dispersing 2.5 mg of the powder and making up to weight with distilled water. Two vials per measuring point were prepared, adjusting the pH manually under magnetic stirring with NaOH and HCl solutions. The dispersions were sonicated after preparation for 1 min to tear the aggregates. Subsequently, they were shaken and left for sedimentation for another 5 min. After this time, the supernatant was extracted, and

the deposit was placed in the stove at 70 °C to estimate the representative amount of sample studied. Afterwards, the pH of the supernatants was checked, and the conductivity was set to 330 μS/cm with NaCl 0.1 M. Samples were shaken vigorously prior to their insertion into the measuring devices. Equilibration time was set to 120 s. Average results obtained from 5 measurements. Optic micrograph analysis was carried out with a Nikon Eclipse Ti-E motorized inverted microscope equipped with objectives: Plan Fluor 10x, Plan apochromatic 20x, 40x, and 60x oil, with a transmission detector. ICP-MS measurements were performed on an XSERIES 2 (Thermo Fisher, Waltham, MA, USA) equipped with a Xt interface option, three-legged torch, Peltier cooled (-15 ºC) glass spray chamber, and PFA-20 nebulizer (operated at an Ar flow of 0.54 L/min). The plasma was operated at 1400 W. Helium, containing 8% of hydrogen, was used as cell reagent gas and oxygen (20% O_2 in Ar) was provided to the extra leg of the three-legged torch option at a flow rate of 255 mL/min.

2 Crystallographic data

X-ray Diffraction Data Collection and Structure Determination. X-ray data collection on a suitable crystal of **GR-MOF-11** to **14** was done at 293(2) K on a Bruker D8 Venture diffractometer using a photon detector equipped with graphite-monochromated MoKα radiation (λ = 0.71073 Å). The data reduction was performed with the APEX3 software[1] and corrected for absorption using SADABS.[2] Crystal structure was solved by direct methods using the SHELXT program[3] and refined by full-matrix least-squares on F2 including all reflections, using anisotropic displacement parameters. All hydrogen atoms were located in difference Fourier maps, and included, as fixed contributions riding on attached atoms with isotropic thermal displacement parameter 1.2 times those of their parent atoms. The OLEX2 software[4] was used as a graphical interface. Lattice water molecules could not be refined due to their disordered disposition in the voids of the structure, so they were removed from the diffraction data by the SQUEEZE procedure implemented in PLATON program[5] during the refinement, but were considered for calculations of the empirical formula, formula weight, density, linear absorption coefficient, and F(000). Crystallographic data for the reported structure have been deposited with the Cambridge Crystallographic Data Center as supplementary CCDC. Copies of the data can be obtained free of charge at http://www.ccdc.cam.ac.uk/products/csd/request.

Table S1. Crystallographic data and structure refinement details of compound **GR-MOF-11**, **GR-MOF-12**, **GR-MOF-13** and **GR-MOF-14**.

**Compound**	**GR-MOF-11**	**GR-MOF-12**	**GR-MOF-13**	**GR-MOF-14**
**Formula**	$[Sr(C_{20}H_{10}N_2O_4)](H_2O)_2$	$[Y(C_{20}H_{10}N_2O_4)_{1.5}](C_3H_7NO)$	$[Cd(C_{20}H_{10}N_2O_4)]_2(C_3H_7NO)(H_2O)_2$	$[Ba(C_{20}H_{10}N_2O_4)](C_3H_7NO)_2$
**Formula weight**	465.95	675.46	1017.55	625.82
**CCDC**	2193840	2193841	2193843	2193842
**Crystal system**	Monoclinic	Triclinic	Monoclinic	Monoclinic
**Space group**	I2/a	P1(2)	$P2_1/n$	P2/c
a **(Å)**	7.9242(14)	7.6744(2)	8.9255(5)	14.5889(4)
b **(Å)**	11.5023(17)	12.9378(3)	13.5861(10)	7.1899(2)
c **(Å)**	18.9113(19)	14.5120(3)	15.3287(11)	25.3467(8)
α **(°)**	90	90.892(2)	90	90
β **(°)**	100.152(5)	104.218(2)	102.731(4)	96.530(2)
γ **(°)**	90	95.547(2)	90	90
**V ($Å^3$)**	1696.71	1389.06	1813.1	2641.44
**Z**	4	2	2	4
**GoF[a]**	1.091	1.034	1.024	1.092
**R_1^b/wR_2^c [I > 2σ(I)]**	0.0321/0.0708	0.0642/0.1551	0.0371/0.0888	0.0400/0.1111
**R_1^b/wR_2^c[all data]**	0.0321/0.0765	0.0642/0.1814	0.0371/0.1003	0.0400/0.1121

3 Selected bond lengths and angles data

Table S2. Table of the selected bond lengths (Å) and angles (°) for compound **GR-MOF-11**.

Bond	Distance	Angle	Degree
Sr1–O1	2.661	O1–Sr1–O2	48.70
Sr1– O2	2.731	O1-Sr-O2(ii)	70.65
Sr1– O1(i)	2.661	O1-Sr-O1(ii)	83.60
Sr1– O2(ii)	2.731	O1-Sr-O2(i)	76.78
Sr1– O2(i)	2.506	O1-Sr-O2(iii)	123.56
Sr1– O1W	2.601	O1-Sr-O1W(ii)	93.06
Sr1– O2(iii)	2.506	O1-Sr-O1W	150.00
Sr1– O1W(ii)	2.601	O2-Sr-O1(ii)	70.65
		O2-Sr-O2(iii)	74.86
		O2-Sr-O2(ii)	96.65
		O2-Sr-O2(i)	123.38
		O2-Sr-O1W(ii)	84.75
		O2-Sr-O1W	155.72
		O1(ii)-Sr-O2(ii)	48.70
		O1(ii)-Sr- O2(iii)	76.78
		O1(ii)-Sr-O2(i)	123.56
		O1(ii)-Sr-O1W	93.06
		O1(ii)-Sr-O1W(ii)	150.00
		O2(ii)-Sr-O2(i)	74.86
		O2(ii)-Sr-O2(iiii)	123.38
		O2(ii)-Sr-O1W	84.75
		O2(ii)-Sr-O1W(ii)	155.72
		O2(i)-Sr-O2(iii)	154.89
		O2(i)-Sr-O1W	80.48
		O2(i)-Sr-O1W(ii)	84.11
		O2(iii)-Sr1- O1W(ii)	80.48

Table S3. Table of the selected bond lengths (Å) and angles (°) for compound **GR-MOF-12**.

Bond	Distance	Angle	Degree
Y1–O1(i)	2.23(3)	O1(i)–Y1–O3(iii)	100(1)
Y1–O3(iii)	2.29(3)	O1(i)–Y1–O18(ii)	80(1)
Y1–O18(ii)	2.42(3)	O1(i)–Y1–O19(ii)	90(1)
Y1–O19 (ii)	2.35(3)	O1(i)–Y1–O27	130(1)
Y1–O27	2.48(3)	O1(i)–Y1–O27(i)	160(1)
Y1–O27(i)	2.30(3)	O1(i)–Y1–O29	77(1)
Y1–O29	2.47(3)	O1(i)–Y1–O40	80(1)
Y1–O40(i)	2.32(3)	O3(iii)–Y1–O18(ii)	156(1)
		O3(iii)–Y1–O19(ii)	148(1)
		O3(iii)–Y1–O27	75(1)
		O3(iii)–Y1–O27(i)	85(1)
		O3(iii)–Y1–O29	83(1)
		O3(iii)–Y1–O40	80(1)
		O18(ii)–Y1–O19(ii)	55(1)
		O18(ii)–Y1–O27	122(1)
		O18(ii)–Y1–O27(i)	87(1)
		O18(ii)–Y1–O29	120(1)
		O18(ii)–Y1–O40	77(1)
		O19(ii)–Y1–O27	75(1)
		O19(ii)–Y1–O27(i)	96(1)
		O19(ii)–Y1–O29	69(1)
		O19(ii)–Y1–O40	132(1)
		O27–Y1– O27(i)	70(1)
		O27–Y1– O29	52.8(9)
		O27–Y1– 40	144(1)
		O27(i)–Y1–O29	123(1)
		O27(i)–Y1–O40	82(1)
		O29–Y1–O40	148(1)

Table S4. Table of the selected bond lengths (Å) and angles (°) for compound **GR-MOF-13**.

Bond	Distance	Angle	Degree
Cd1–N11	2.324(4)	N26–Cd1–N11	71.8(2)
Cd1–N26	2.359(5)	N26–Cd1–O18	93.7(1)
Cd1–O1	2.271(3)	N26–Cd1–O3	171.6(1)
Cd1–O19	2.527(4)	N26–Cd1–O19	96.9(1)
Cd1–O18	2.296(4)	N26–Cd1–O1	80.4(1)
Cd1–O3	2.290(4)	N11–Cd1–O1	99.6(1)
		N11–Cd1–O3	112(1)
		N11–Cd1–O18	130.6(1)
		N11–Cd1–O19	80.2(1)
		O1–Cd1–O3	91.5(1)
		O1–Cd1–O18	124.9(1)
		O1–Cd1–O19	177.2(1)
		O3-Cd1-O19	91.1(1)
		O3-Cd1-O18	89.1(1)
		O18-Cd1-O19	54.2(1)

Table S5. Table of the selected bond lengths (Å) and angles (°) for compound **GR-MOF-14**.

Bond	Distance	Angle	Degree
Ba1– O1	2.710(4)	O1–Ba1–O2(i)	134.9(1)
Ba1–O2(i)	2.712(3)	O1–Ba1–O14	75.5(1)
Ba1– O14	2.718(4)	O1–Ba1–O14(ii)	98.5(1)
Ba1– O14(ii)	2.831(3)	O1–Ba1–O15(ii)	75.6(1)
Ba1– O15(ii)	2.959(3)	O1–Ba1–O15(iii)	94.0(1)
Ba1– O15(iii)	2.729(4)	O1–Ba1–O27	148.4(1)
Ba1– O27	2.752(4)	O1–Ba1–O31	76.1(1)
Ba1– O31	2.754(5)	O2(i)–Ba1–O14	130.26
		O2(i)–Ba1–O14(ii)	68.4(1)
		O2(i)–Ba1–O15(ii)	64.8(1)
		O2(i)–Ba1–O15(iii)	65.4(1)
		O2(i)–Ba1–O27	75.5(1)
		O2(i)–Ba1–O31	134.1(1)
		O14-Ba1-O14(ii)	68.8(9)
		O14-Ba1-O15(ii)	99.9(9)
		O14-Ba1-O15(iii)	164.3(1)
		O14-Ba1-O27	76.3(1)
		O14-Ba1-O31	83.5(1)
		O14(ii)-Ba1-O15(ii)	45.01(9)
		O14(ii)-Ba1-O15(iii)	125.2(1)
		O14(ii)-Ba1-O27	84.3(1)
		O14(ii)-Ba1-O31	152.2(1)
		O15(ii)–Ba1–O15(iii)	88.52(9)
		O15(ii)–Ba1–O27	123.5(1)
		O15(ii)–Ba1–O31	149.6(1)
		O15(iii)–Ba1–O27	110.2(1)
		O15(iii)–Ba1–O31	82.6(1)
		O27-Ba1-O31	86.8(1)

4 PXRD

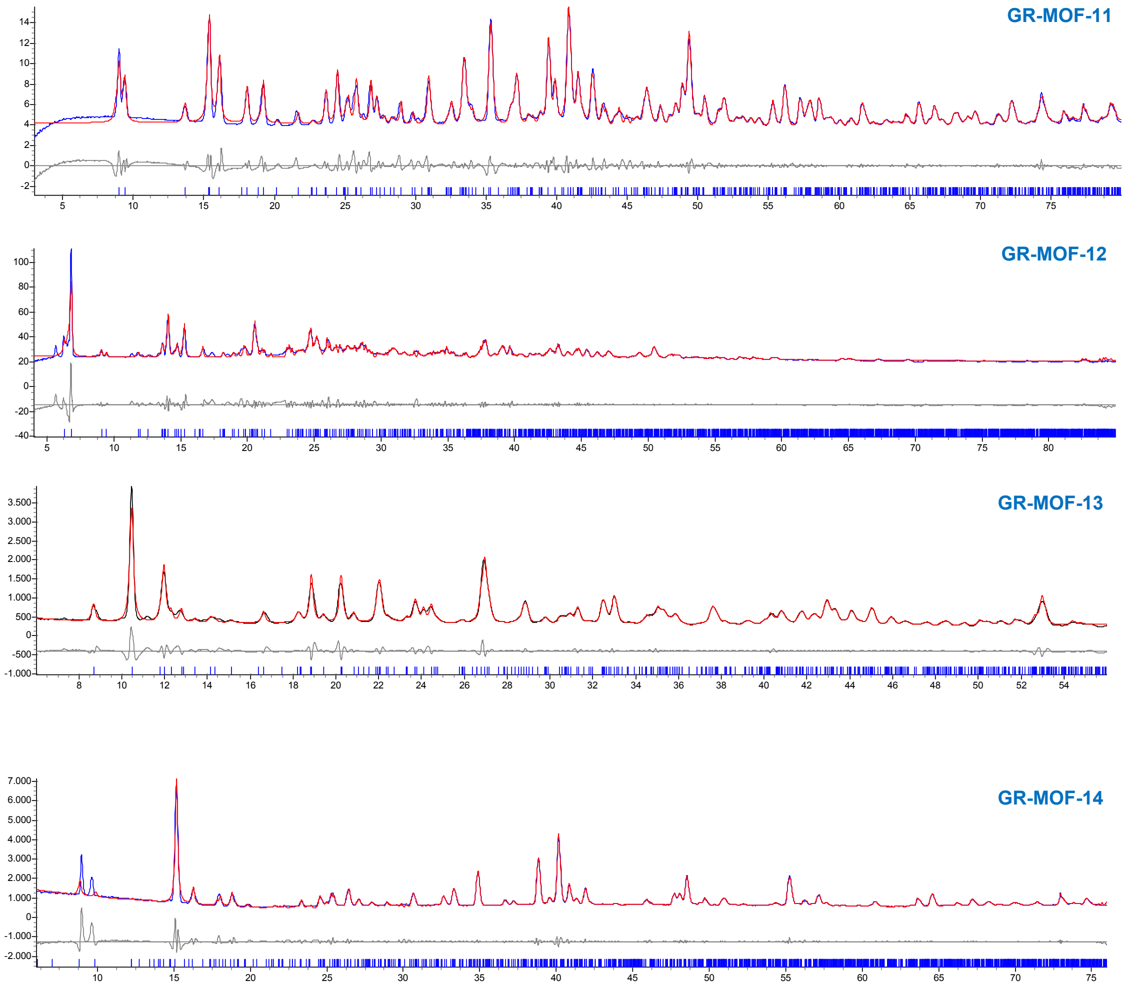

Figure S1. Le Bail fitting of **GR-MOF-11 to 14**.

5 FTIR characterization.

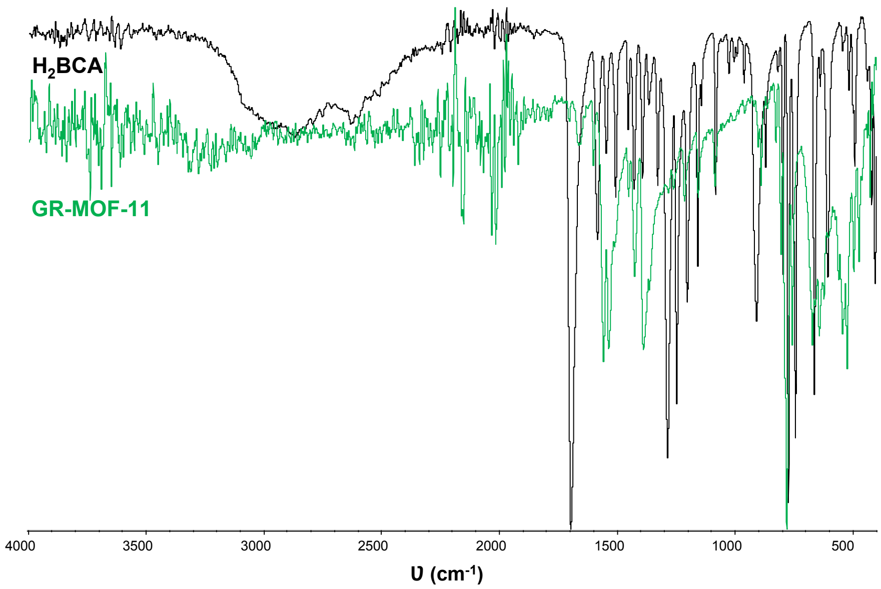

Figure S2. FTIR spectra of **GR-MOF-11** (green) and linker (black).

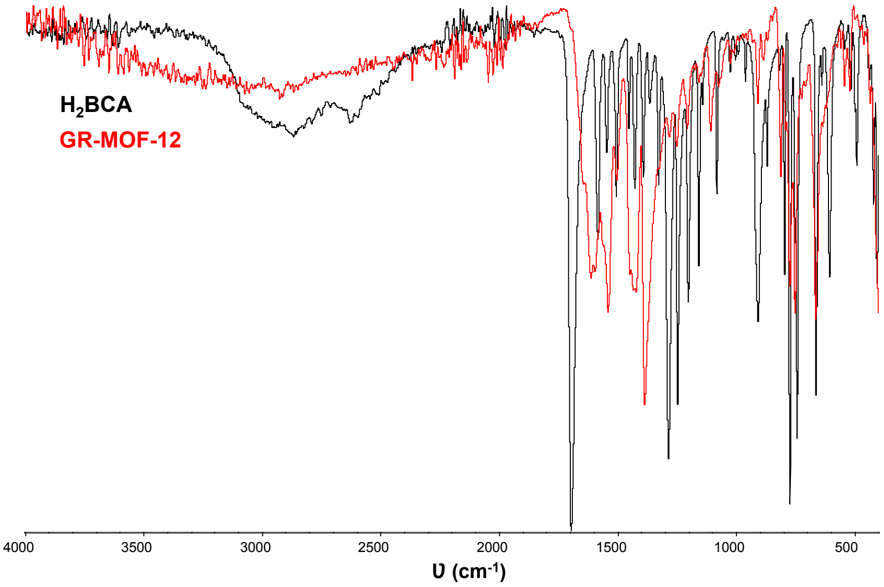

Figure S3. FTIR spectra of **GR-MOF-12** (red) and linker (black).

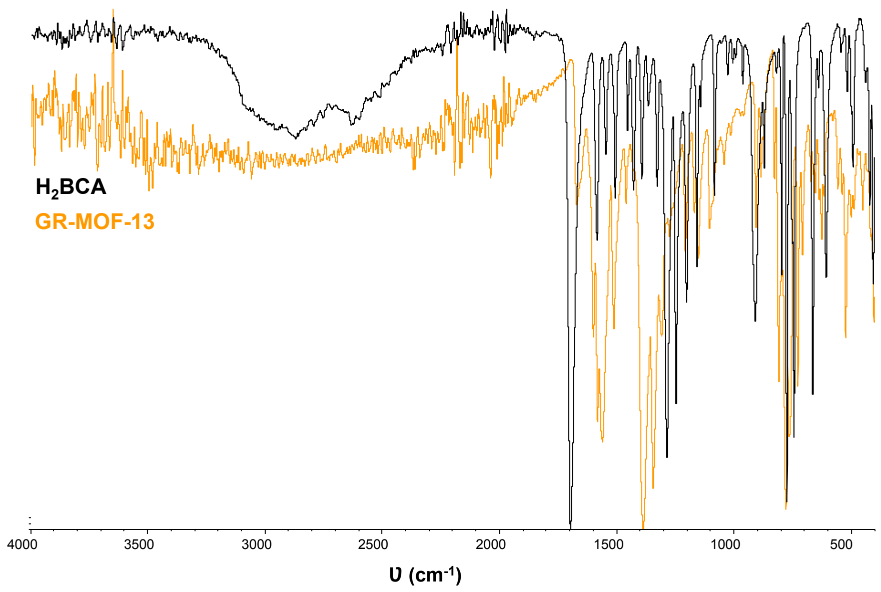

Figure S4. FTIR spectra of **GR-MOF-13** (orange) and linker (black).

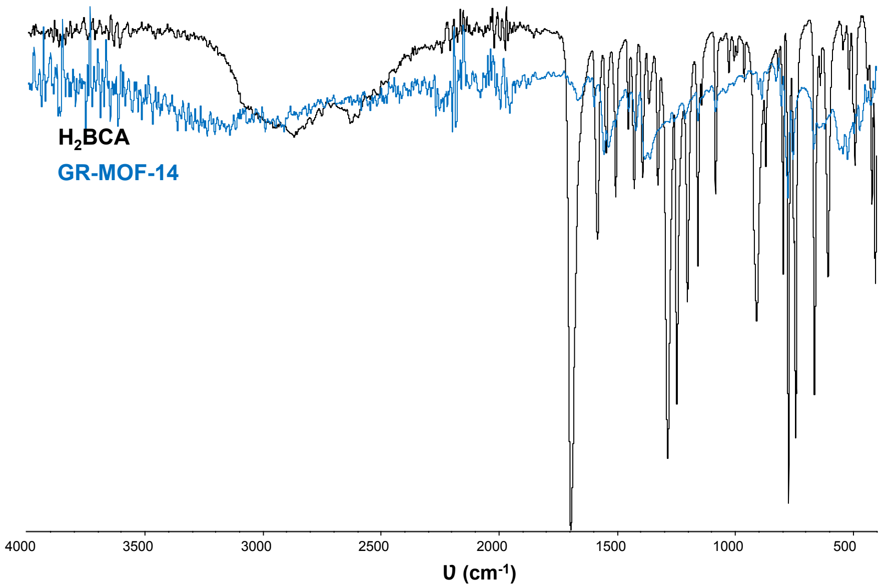

Figure S5. FTIR spectra of **GR-MOF-14** (blue) and linker (black).

6 Thermogravimetric analyses (TGA)

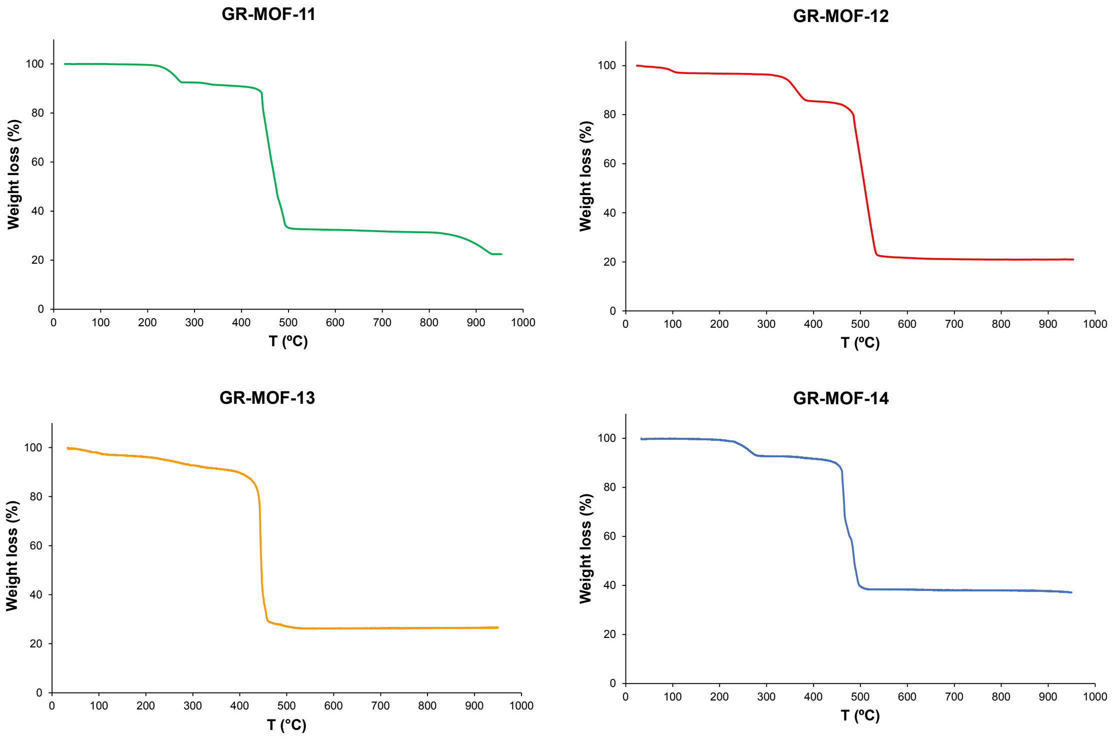

Figure S6. TGA of **GR-MOF-11** to **14**.

7 Photoluminescence measurements

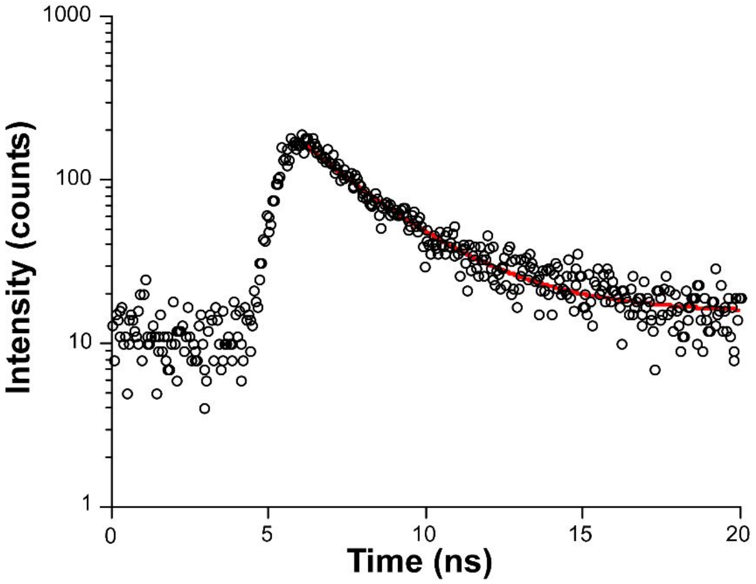

Figure S7. Decay curve (circles) and tail-fitting (red line) to estimate the lifetime of H_2BCA ligand.

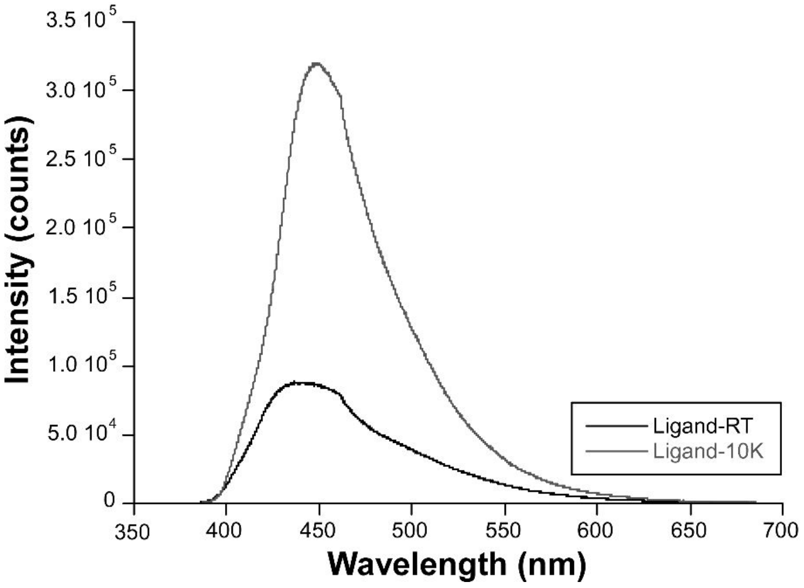

Figure S8. Variable temperature comparative emission of H_2BCA ligand.

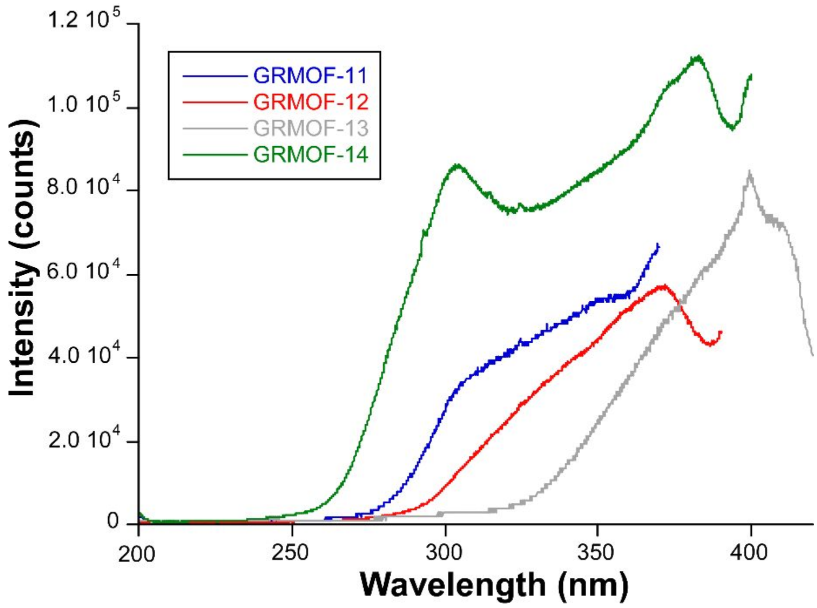

Figure S9. Excitation spectra of **GR-MOFs** recorded at the main emission lines.

The decay curves for the ligand's sample and MOFs were collected at the main emission lines using a pulsed LED PLS-340 (λ = 340 nm). Particularly for **GRMOF-13** a μF900 pulsed lamp was employed to measure the long lifetime associated with the emission at 550 nm.

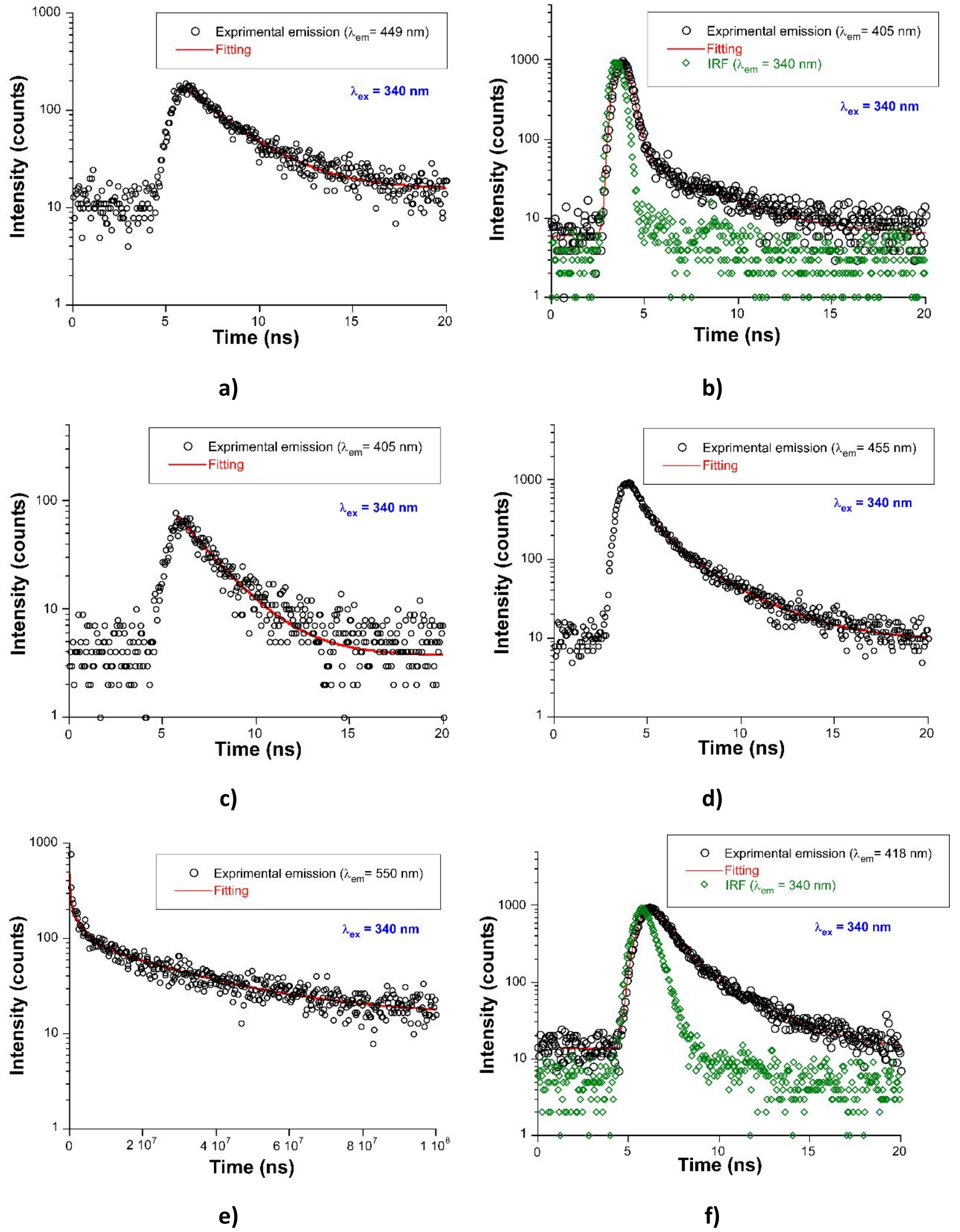

Figure S10. Decay curves measured showing their best fitting for the most relevant emission bands of: a) H_2BCA linker, b) **GR-MOF-11**, c) **GR-MOF-12**, d) **GR-MOF-13** at the emission maximum, e) **GR-MOF-13** at the emission tail, and f) **GR-MOF-14**.

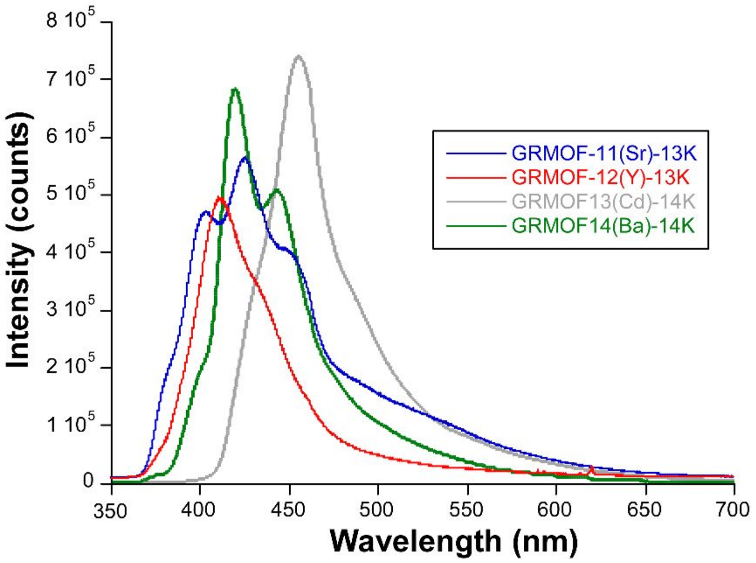

Figure S11. Emission spectra of **GR-MOFs** recorded at low temperature.

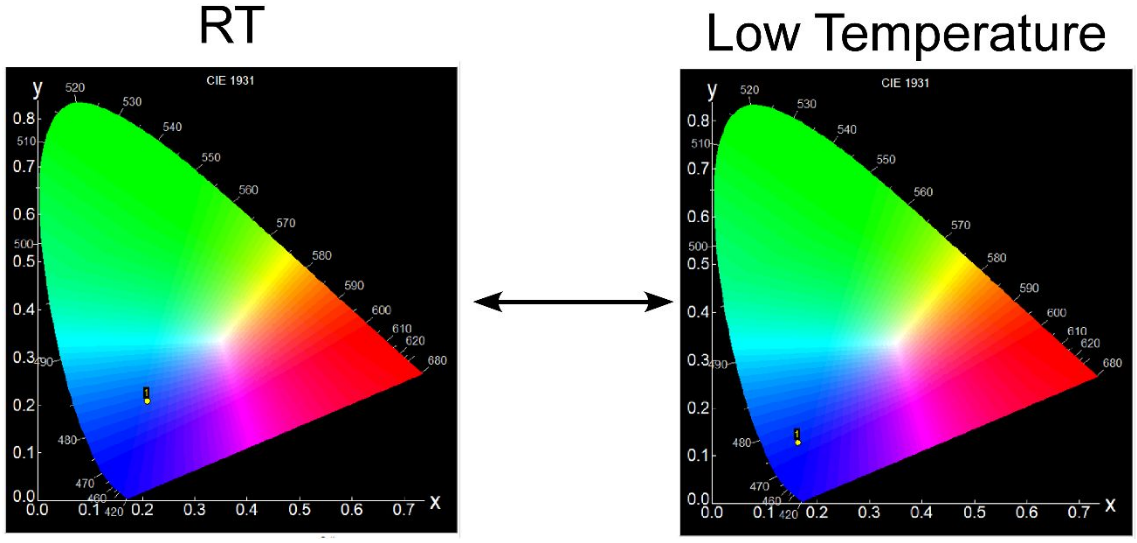

Figure S12. Thermoluminescence observed for **GR-MOF-13** showing the integrated emitted color.

Computational details. Computational calculations were carried out to study the photoluminescence properties from the theoretical point of view. The calculations were performed on the ligand molecule taken from the X-ray coordinates of **GRMOF-11** (named model 1, hereafter). Model 1 was previously optimized with the Gaussian 16

package[6] using the Becke three parameter hybrid functional with the non-local correlation functional of Lee–Yang–Parr (B3LYP)[78] along with the 6-31G++(d,p) basis set.[9] A frequency calculation was also conducted to confirm that an energy minimum was achieved. PL spectra were calculated by means of TD-DFT methodology, computing the 150 lowest excitation states. The results were analyzed using the GaussSum program package[10] and molecular orbitals were plotted using GaussView 6.[11]

8 Particle size distribution of catalysts

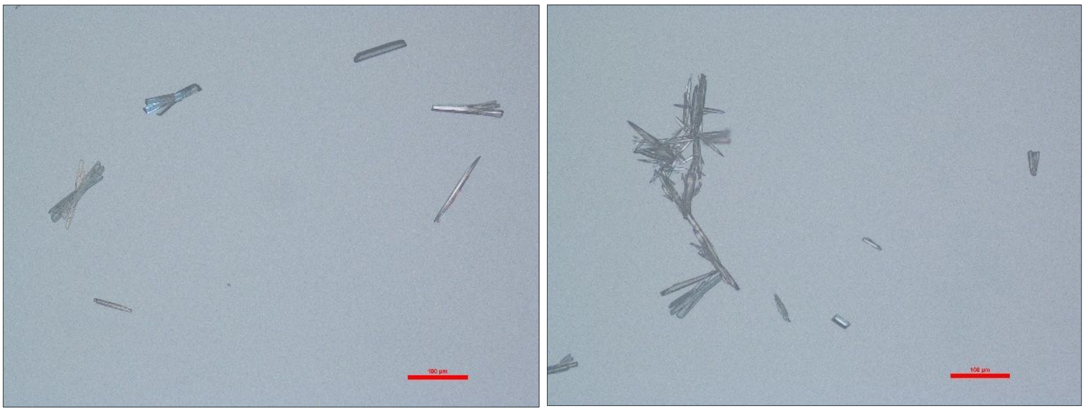

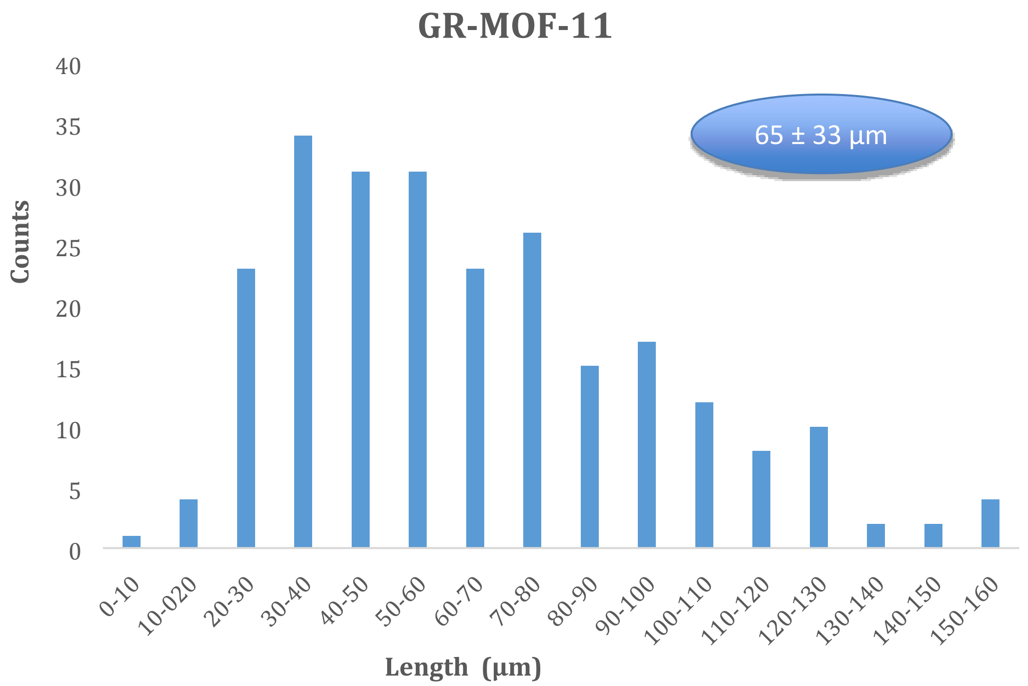

Figure S13. Images and particle size distribution (an overall of 250 particles) in the **GR-MOF-11** catalyst determined from optical microscope images.

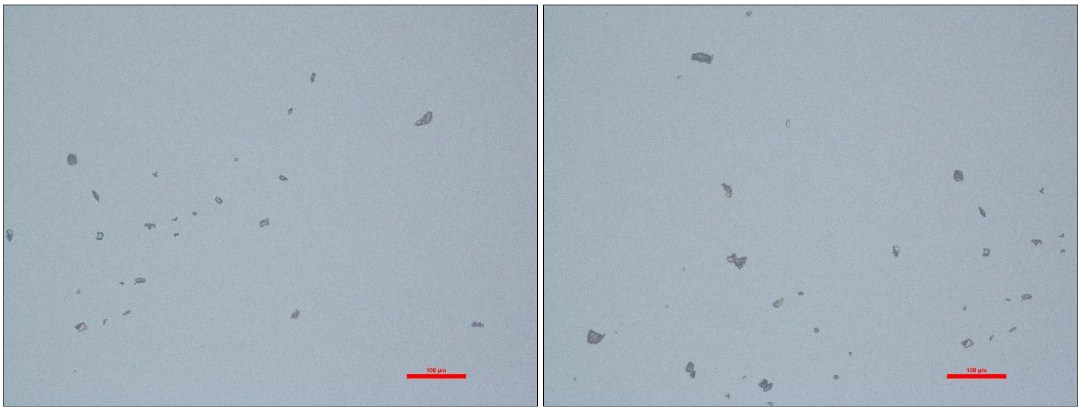

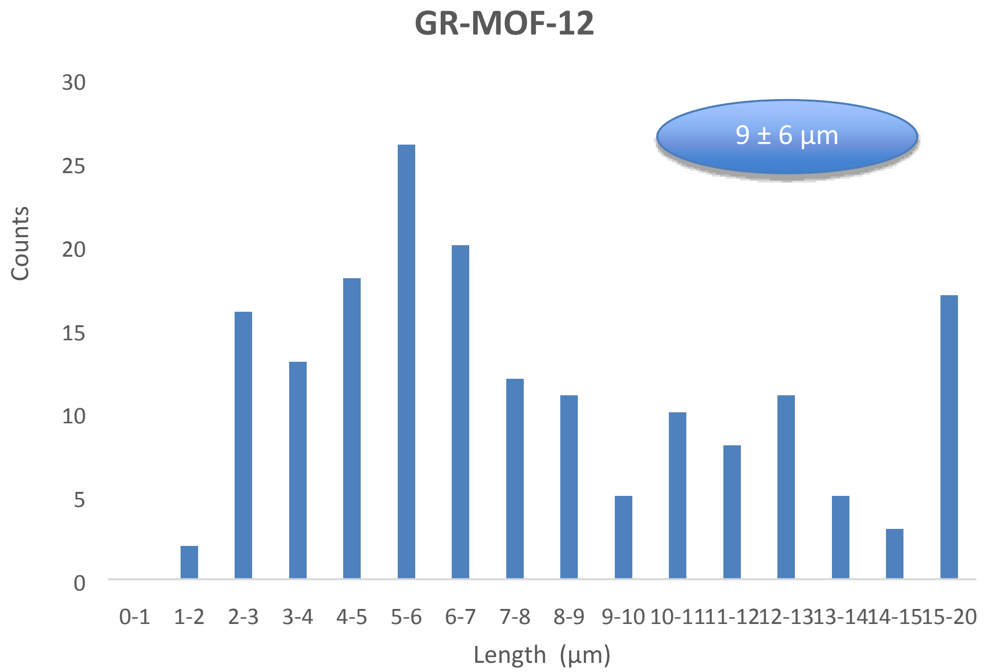

Figure S14. Images and particle size distribution (an overall of 250 particles) in the **GR-MOF-12** catalyst determined from optical microscope images.

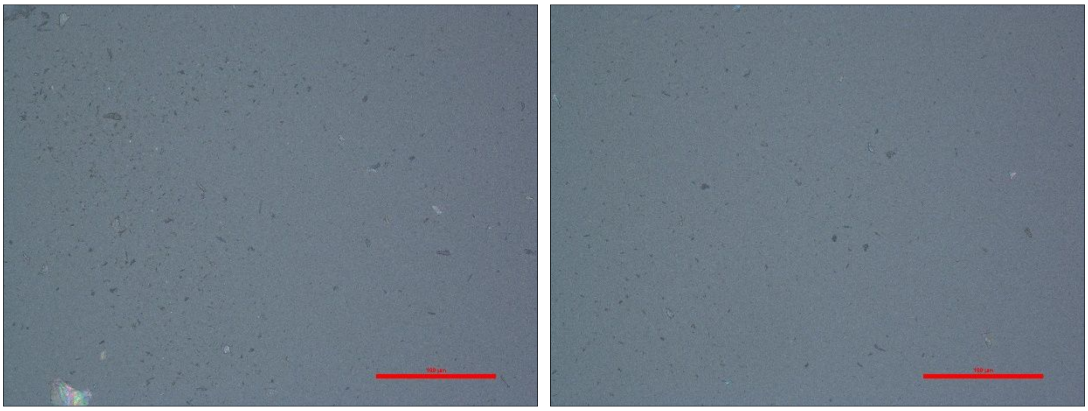

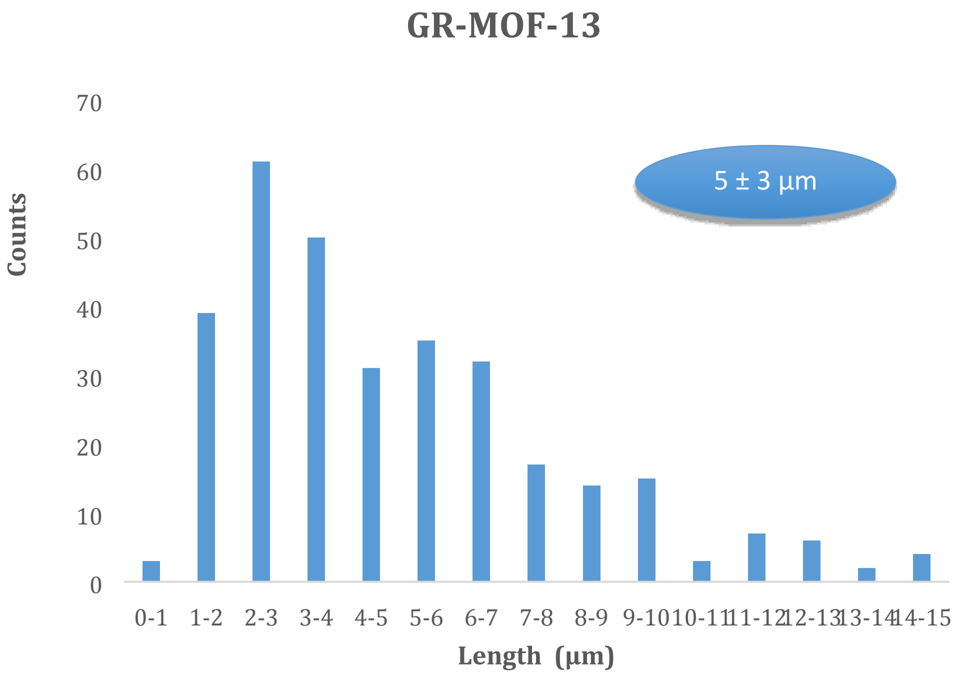

Figure S15. Images and particle size distribution (an overall of 250 particles) in the **GR-MOF-13** catalyst determined from optical microscope images.

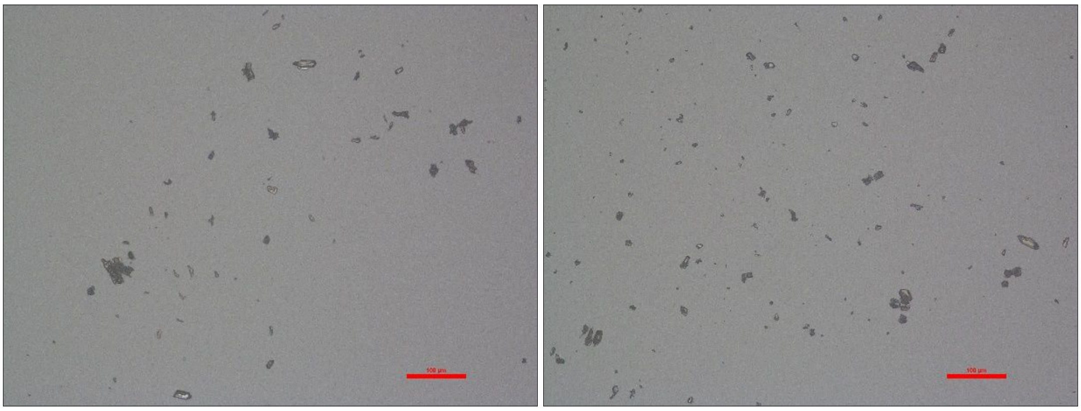

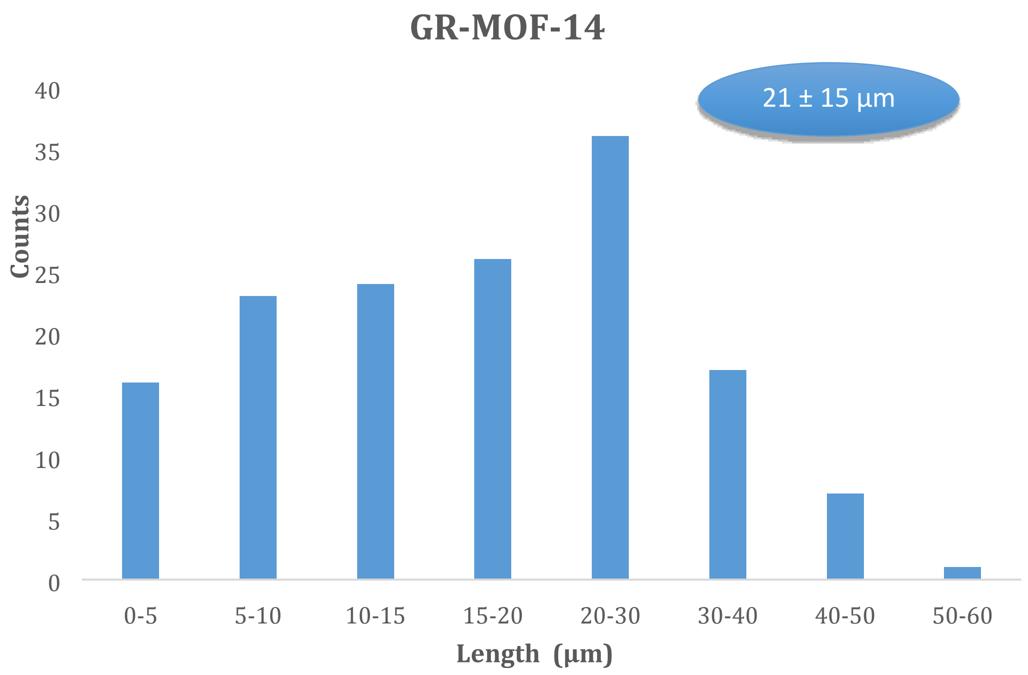

Figure S16. Images and particle size distribution (an overall of 250 particles) in the **GR-MOF-14** catalyst determined from optical microscope images.

9 Study of ζ-potential at different pH values

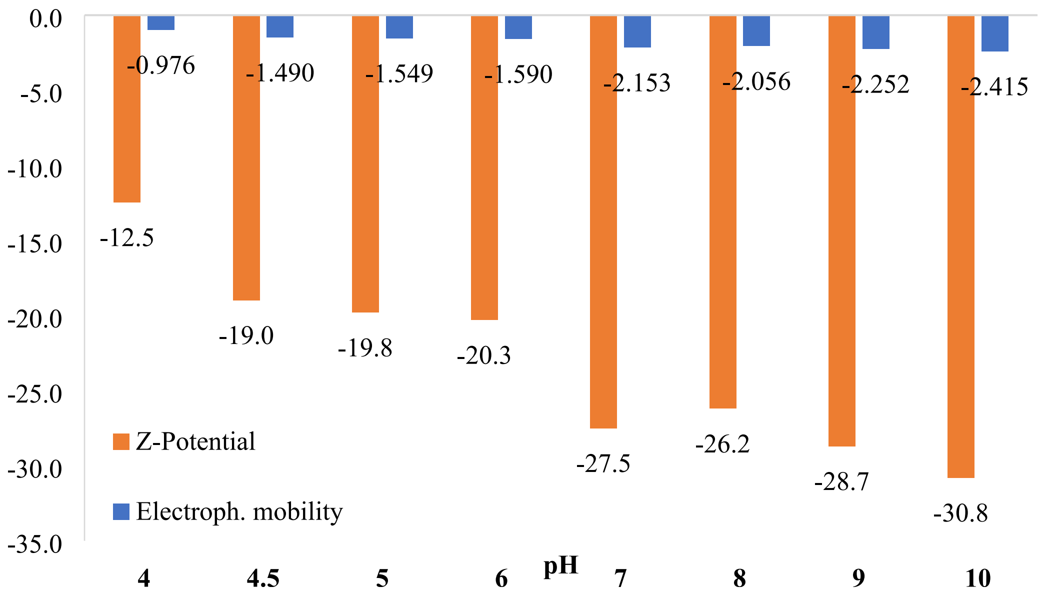

Figure S17. Electrophoretic mobility (µm·cm/V·s) and ζ-potential (mV) dependence with the pH of the **GR-MOF-11.** All the measurements were performed with constant conductivity of 330 µS/cm.

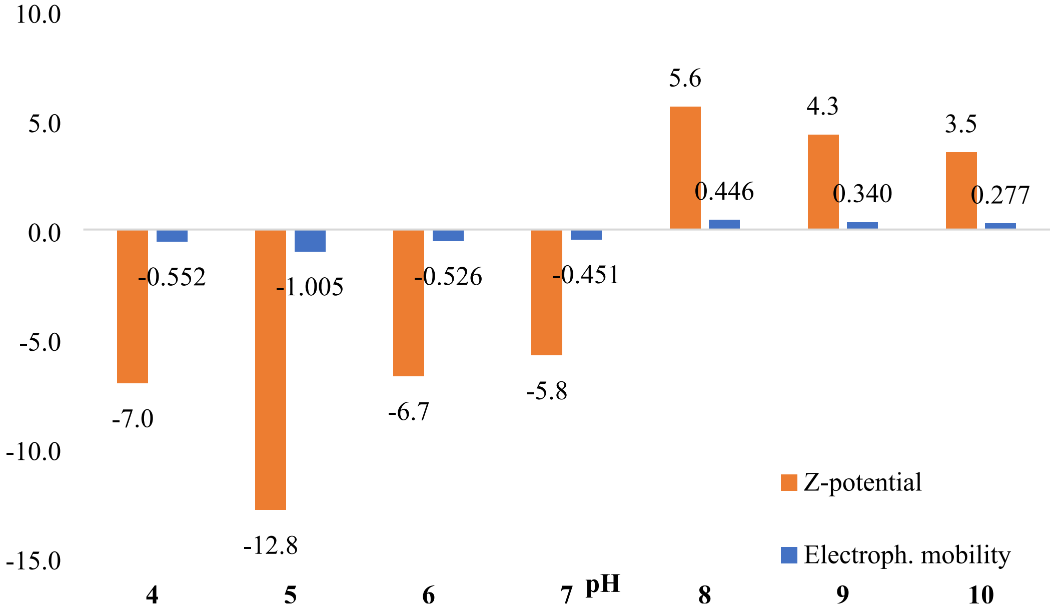

Figure S18. Electrophoretic mobility (µm·cm/V·s) and ζ-potential (mV) dependence with the pH of the **GR-MOF-12.** All the measurements were performed with constant conductivity of 330 µS/cm.

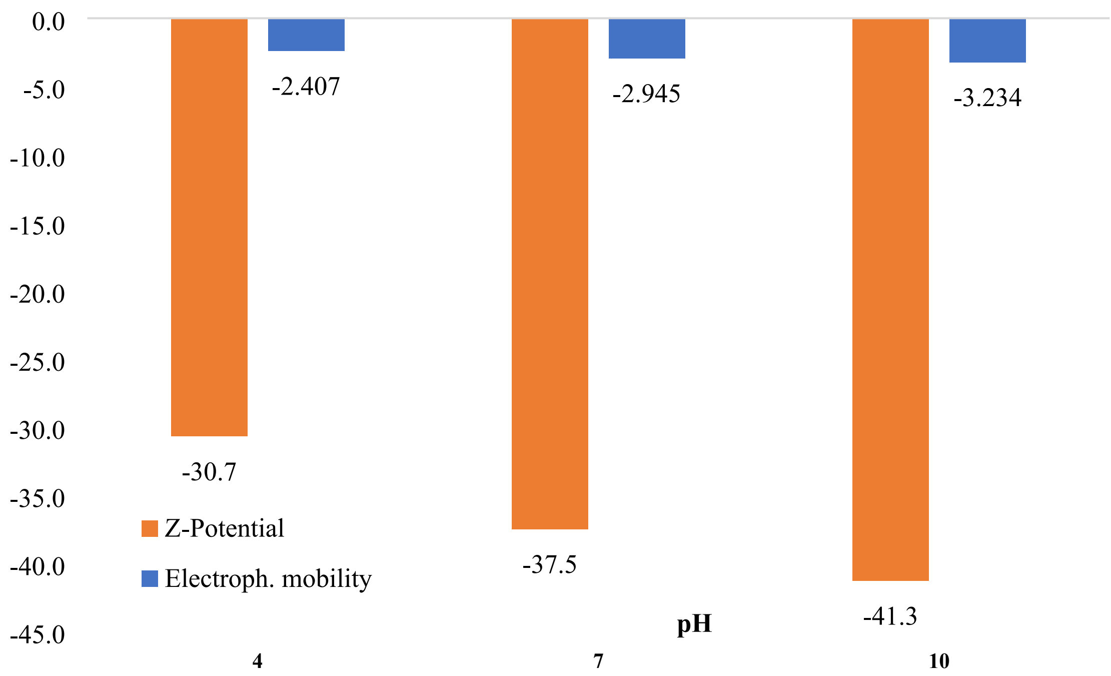

Figure S19. Electrophoretic mobility (µm·cm/V·s) and ζ-potential (mV) dependence with the pH of the **GR-MOF-13.** All the measurements were performed with constant conductivity of 330 µS/cm.

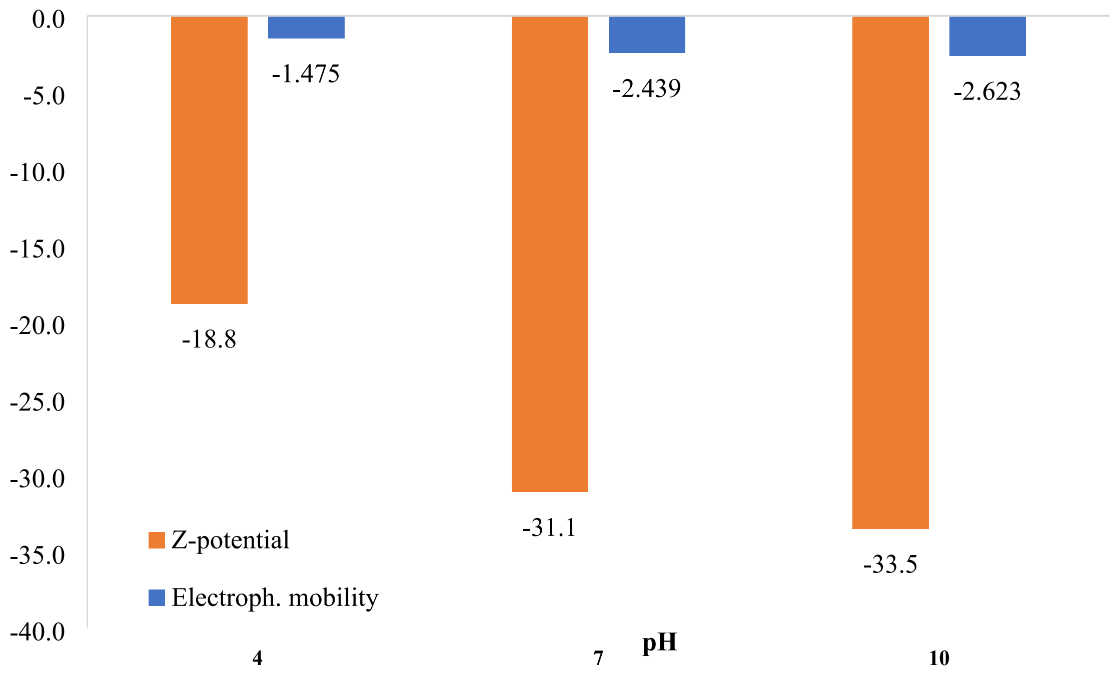

Figure S20. Electrophoretic mobility (µm·cm/V·s) and ζ-potential (mV) dependence with the pH of the **GR-MOF-14.** All the measurements were performed with constant conductivity of 330 µS/cm.

Table S6. Electrophoretic mobility (EM) and ζ-potential dependence with the pH of the **GR-MOFs** particles dispersed in water. Conductivity fixed at 330 µS/cm.

	**GR-MOF-11**		**GR-MOF-12**		**GR-MOF-13**		**GR-MOF-14**	
**pH**	**EM (µmcm/Vs)**	**ζ-potential (mV)**	**EM (µmcm/Vs)**	**ζ-potential (mV)**	**EM (µmcm/Vs)**	**ζ-potential (mV)**	**EM (µmcm/Vs)**	**ζ -potential (mV)**
**4**	0.032 ± 0.027	-12.5 ± 0.8	-2.836 ± 0.117	-36.2 ± 1.5	-2.407 ± 0.046	-30.7 ± 0.6	-1.475 ± 0.073	-18.8 ± 0.9
**4.5**	-1.490 ± 1.112	-19.0 ± 1.5			-	-	-	-
**5**	-1.549 ± 0.067	-19.8 ± 0.8			-	-	-	-
**6**	-1.590 ± 0.060	-20.3 ± 0.8			-	-	-	-
**7**	-2.153 ± 0.048	-27.5 ± 0.6	-2.444 ± 0.094	-31.2 ± 1.2	-2.945 ± 0.064	-37.5 ± 0.8	-2.439 ± 0.073	-31.1 ± 0.9
**8**	-2.056 ± 0.030	-26.2 ± 0.4			-	-	-	-
**9**	-2.252 ± 0.093	-28.7 ± 1.2			-	-	-	-
**10**	-2.415 ± 0.086	-30.8 ± 1.1	-2.752 ± 0.139	-35.1 ± 1,8	-3.234 ± 0.042	-41.3 ± 0.5	-2.623 ± 0.070	-33.5 ± 0.9

10 TOF of GR-MOFs

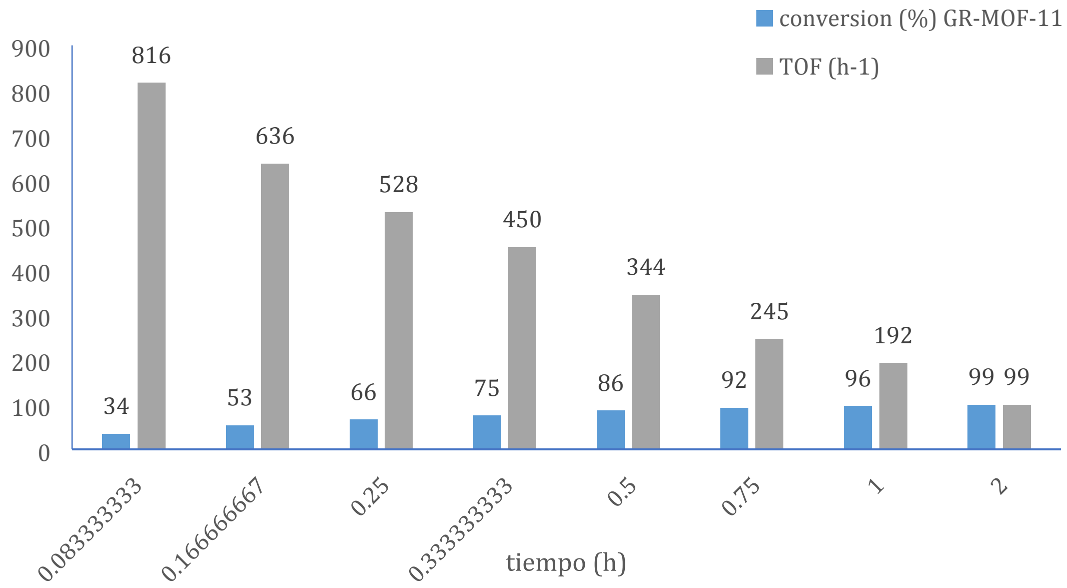

Figure S21. Analysis of the TOF (h^{-1}) obtained at different times of reaction with **GR-MOF-11** (0.5 mol%) using compound **1a** as substrate with the optimized reaction conditions.

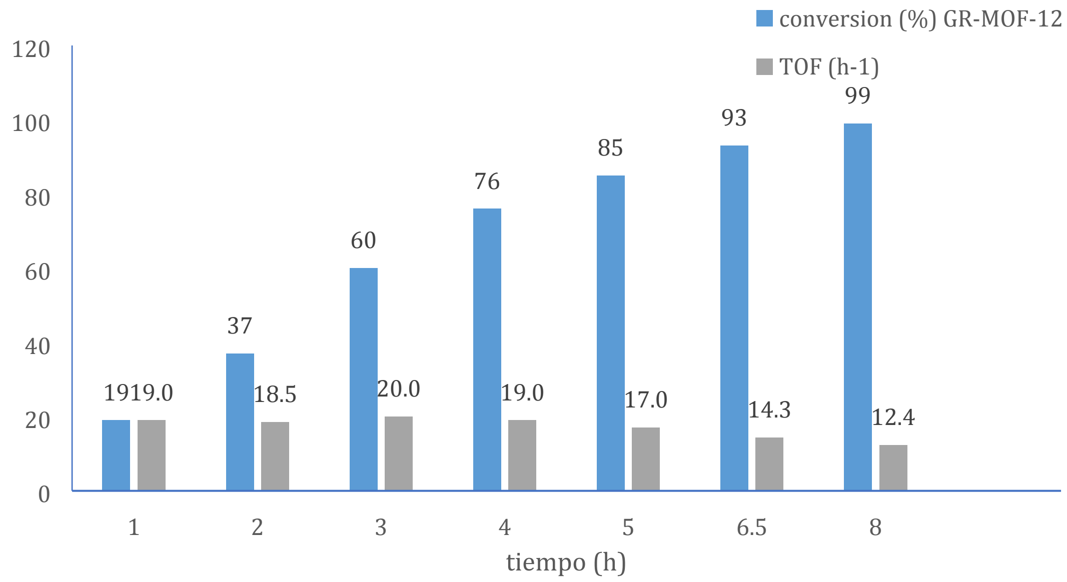

Figure S22. Analysis of the TOF (h^{-1}) obtained at different times of reaction with **GR-MOF-12** (1 mol%) using compound **1a** as substrate with the optimized reaction conditions.

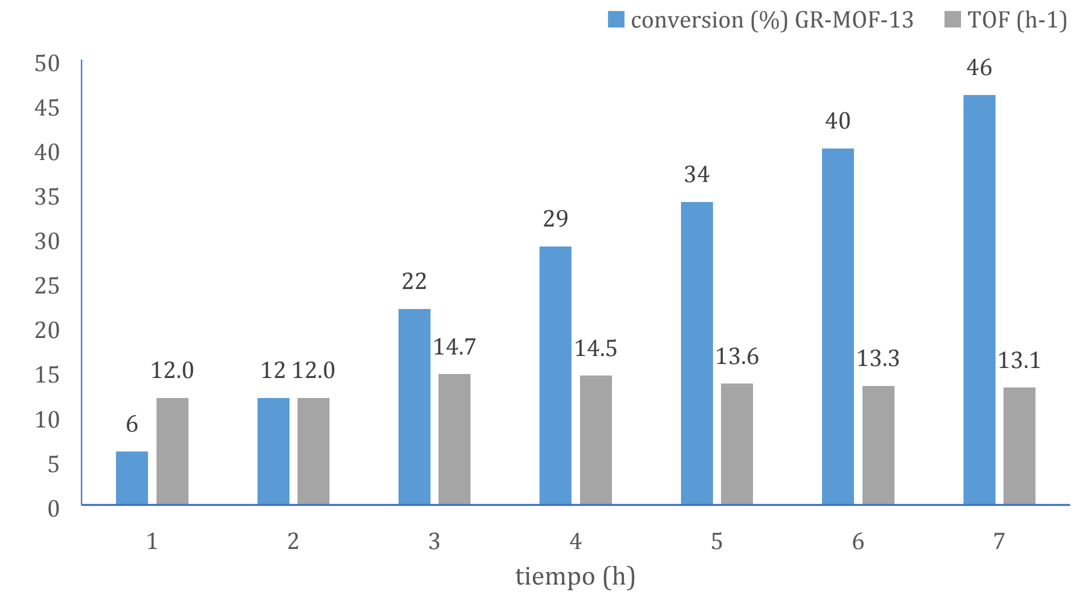

Figure S23. Analysis of the TOF (h^{-1}) obtained at different times of reaction with **GR-MOF-13** (0.5 mol%) using compound **1a** as substrate with the optimized reaction conditions.

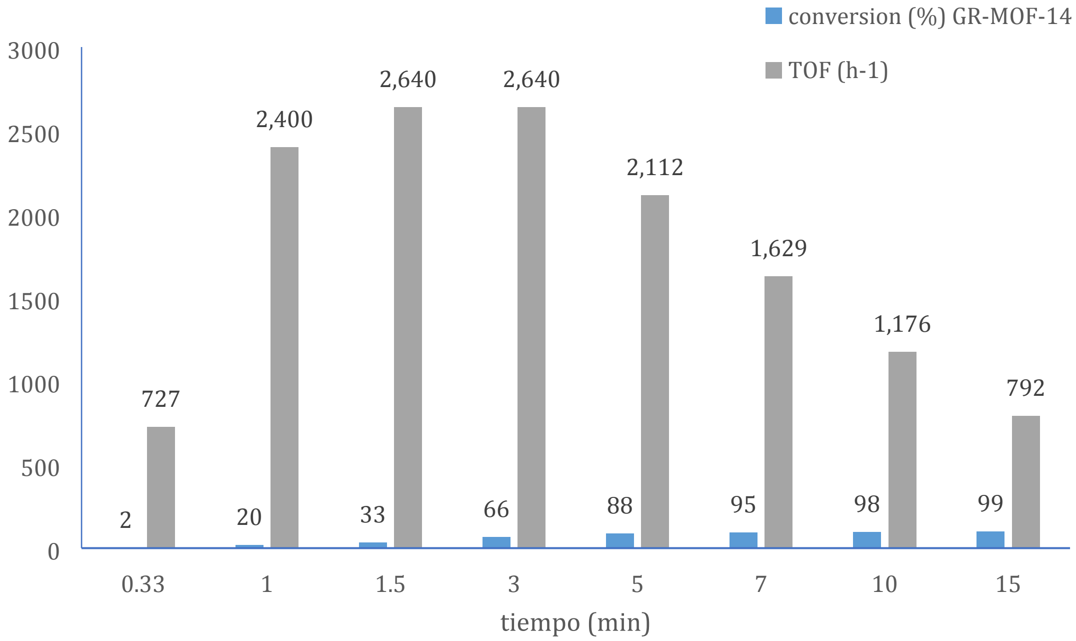

Figure S24. Analysis of the TOF (h^{-1}) obtained at different times of reaction with **GR-MOF-14** (0.5 mol%) using compound **1a** as substrate with the optimized reaction conditions.

11 Green chemistry metrics

Table S7. Green metrics calculated for **GR-MOFs** catalysts.

Sample	AE (Atom Economy)	MI (Mass Intensity)	RME (Reaction Mass Efficiency)	CE (Carbon Efficiency)
**GR-MOF-11**	99.9	1.059	95.4	96.5
**GR-MOF-12**	99.9	1.065 (0.5 mol%)/1.081 (1 mol%)	95.4	96.5
**GR-MOF-13**	99.9	1.061	95.4	96.5
**GR-MOF-14**	99.9	1.064	95.4	96.5

12 Characterization Data of Products

Multiplicity is reported with the usual abbreviations (s: singlet, bs: broad singlet, d: doublet, dd: doublet of doublets, ddd: doublet of doublet of doublets, t: triplet, td: triplet of doublets, q: quartet, dq: doublet of quartet, p: pentet, sex: sextet, hept: heptet, m: multiplet).

2-Phenyl-2-((trimethylsilyl)oxy)acetonitrile (2a). This product has been previously reported.[12] ^{1}H NMR (300.13 MHz, $CDCl_3$): δ 7.50–7.35 (m, 5H, ArH), 5.50 (s, 1H, *CH*CN), 0.23 (s, 9H, TMS) ppm. ^{13}C NMR (75.48 MHz, $CDCl_3$): δ 136.2 (C_{ipso}), 129.3(ArCH), 128.9 (ArCH), 126.3 (ArCH), 119.1 (CN), 63.6 (CH), -0.29 (TMS) ppm.

2-(4-Methoxyphenyl)-2-((trimethylsilyl)oxy)acetonitrile (2b). This product has been previously reported.[13] ^{1}H NMR (300.13 MHz, $CDCl_3$): δ 7.38 (d, J = 8.6 Hz, 2H, ArH), 6.92 (d, J = 8.6 Hz, 2H, ArH), 5.43 (s, 1H, *CH*CN), 3.82 (s, 3H, OMe), -0.21 (s, 9H, TMS) ppm. ^{13}C NMR (75.48 MHz, $CDCl_3$): δ 160.3 (C_{ipso}), 128.3 (C_{ipso}), 127.9 (ArCH), 119.3 (CN), 114.2 (ArCH), 63.3 (CH), 55.3 (OCH_3), -0.24 (TMS) ppm.

2-(4-Chlorophenyl)-2-((trimethylsilyl)oxy)acetonitrile (2c). This product has been previously reported.[14] ^{1}H NMR (300.13 MHz, $CDCl_3$): δ 7.45–7.40 (m, 5H, ArH), 5.49 (s, 1H, *CH*CN), 0.26 (s, 9H, TMS) ppm. ^{13}C NMR (75.48 MHz, $CDCl_3$): δ 135.3 (C_{ipso}), 134.8 (C_{ipso}), 129.1 (ArCH), 127.7 (ArCH), 118.8 (CN), 63.0 (CH), -0.30 (TMS) ppm.

2-(Pyridin-2-yl)-2-((trimethylsilyl)oxy)acetonitrile (2d). This product has been previously reported.[15] ^{1}H NMR (300.13 MHz, $CDCl_3$): δ 8.60–8.55 (m, 1H, ArH), 7.79 (dt, J = 7.7, 1.7 Hz, 1H, ArH), 7.59 (d, J = 7.7 Hz, 1H, ArH), 7.35–7.25 (m, 1H, ArH), 5.58 (s, 1H, *CH*CN), 0.26 (s, 9H, TMS) ppm. ^{13}C NMR (75.48 MHz, $CDCl_3$): δ 155.4 (C_{ipso}), 149.3 (ArCH), 137.5(ArCH), 124.0 (ArCH), 120.5 (ArCH), 118.6 (CN), 65.1 (CH), -0.37 (TMS) ppm.

2-((Trimethylsilyl)oxy)butanenitrile (2e). This product has been previously reported.[16] ^{1}H NMR (300.13 MHz, $CDCl_3$): δ 4.34 (t, J = 6.3 Hz, 1H, CH), 1.85–1.75 (m, 2H, CH_2), 1.04 (t, J = 7.4 Hz, 3H, CH_3), 0.21 (s, 9H, CH_3 x 3) ppm. ^{13}C NMR (75.48 MHz, $CDCl_3$): δ 119.9 (CN), 62.7 (CH), 29.6 (CH_2), 8.9 (CH_3), 0.4 (TMS) ppm.

2-Phenyl-2-((trimethylsilyl)oxy)propanenitrile (2f). This product has been previously reported.[17] ^{1}H NMR (300.13 MHz, $CDCl_3$): δ 7.60–7.50 (m, 2H, ArH), 7.45–7.30 (m, 3H, ArH), 1.86 (s, 3H, CH_3), 0.18 (s, 9H, TMS) ppm. ^{13}C NMR (75.48 MHz, $CDCl_3$): δ 142.0 (C_{ipso}), 128.68 (ArCH), 128.66 (ArCH), 124.6 (ArCH), 121.6 (CN), 71.6 (C), 33.5 (CH_3), 1.03 (TMS) ppm.

2-(4-Methoxyphenyl)-2-((trimethylsilyl)oxy)propanenitrile (2g). This product has been previously reported.[18] ^{1}H NMR (300.13 MHz, $CDCl_3$): δ 7.46 (d, *J* = 8.7 Hz, 2H, ArH), 6.91 (d, *J* = 8.7 Hz, 2H, ArH), 3.82 (s, 3H, OMe), 1.85 (s, 3H, CH_3), 0.16 (s, 9H, TMS) ppm. ^{13}C NMR (75.48 MHz, $CDCl_3$): δ 159.7 (C_{ipso}), 133.9 (C_{ipso}), 126.0 (ArCH), 121.7 (CN), 113.8 (ArCH), 71.2 (C), 55.2 (OCH_3), 33.3 (CH_3), 1.00 (TMS) ppm.

2-(4-Chlorophenyl)-2-((trimethylsilyl)oxy)propanenitrile (2h). This product has been previously reported.[18] ^{1}H NMR (300.13 MHz, $CDCl_3$): δ 7.48 (d, *J* = 8.5 Hz, 2H, ArH), 7.37 (d, *J* = 8.5 Hz, 2H, ArH), 1.83 (s, 3H, CH_3), 0.19 (s, 9H, TMS) ppm. ^{13}C NMR (75.48 MHz, $CDCl_3$): δ 140.6 (C_{ipso}), 134.5 (C_{ipso}), 128.8 (ArCH), 126.0 (ArCH), 121.2 (CN), 71.0 (C), 33.4 (CH_3), 1.00 (TMS) ppm.

2-(Pyridin-2-yl)-2-((trimethylsilyl)oxy)propanenitrile (2i). This product has been previously reported.[19] ^{1}H NMR (300.13 MHz, $CDCl_3$): δ 8.62 (d, *J* = 4.7 Hz, 1H, ArH), 7.77 (t, *J* = 7.8 Hz, 1H, ArH), 7.60 (d, J = 7.8 Hz, 1H, ArH), 7.30–7.25 (m, 1H, ArH), 1.93 (s, 3H, CH_3), 0.26 (s, 9H, TMS) ppm. ^{13}C NMR (75.48 MHz, $CDCl_3$): δ 160.0 (C_{ipso}), 149.0 (ArCH), 137.2 (ArCH), 123.4 (ArCH), 121.3 (CN), 118.9 (ArCH), 72.9 (C), 31.2 (CH_3), 1.06 (TMS) ppm.

2-Methyl-2-((trimethylsilyl)oxy)butanenitrile (2j). This product has been previously reported.[18] ^{1}H NMR (300.13 MHz, $CDCl_3$): δ 1.85–1.65 (m, 2H, CH_2), 1.55 (s, 3H, CH_3), 1.04 (t, *J* = 7.4 Hz, 3H, CH_3CH_2), 0.23 (s, 9H, TMS) ppm. ^{13}C NMR (75.48 MHz, $CDCl_3$): δ 121.9 (CN), 70.2 (C), 36.4 (CH_2), 28.4 (CH_3), 8.6 (CH_3), 1.21 (TMS) ppm.

2-(2,4-Difluorophenyl)-2-((trimethylsilyl)oxy)propanenitrile (**2k**). This product has been previously reported.[20] ^{1}H NMR (500.13 MHz, $CDCl_3$): δ 7.56 (td, *J* = 8.8, 6.4 Hz, 1H, ArH), 6.95–6.90 (m, 1H, ArH), 6.86 (ddd, *J* = 11.1, 8.8, 2.5 Hz, 1H, ArH), 1.92 (s, 3H, CH_3), 0.27 (s, 9H, TMS) ppm. ^{13}C NMR (125.77 MHz, $CDCl_3$): δ 163.2 (dd, *J* = 250.8, 12.0 Hz, C_{ipso}-F), 159.4 (dd, *J* = 252.5, 12.0 Hz, C_{ipso}-F), 127.8 (dd, *J* = 9.7, 4.3 Hz, ArCH), 125.0 (dd, *J* = 11.2, 3.9 Hz, C_{ipso}-F), 120.4 (CN), 111.2 (d, *J* = 21.2 Hz, ArCH), 104.9 (t, *J* = 25.6 Hz, ArCH), 68.0 (d, *J* = 2.0 Hz, C), 30.8 (d, *J* = 2.9 Hz, CH_3), 1.08 (TMS) ppm.

1-Phenylethan-1-ol (**4a**). This product has been previously reported.[21] ^{1}H NMR (300.13 MHz, $CDCl_3$): δ 7.30–7.20 (m, 5H, ArH), 4.82 (q, *J* = 6.5 Hz, 1H, CH), 1.83 (br s, 1H, OH), 1.42 (d, *J* = 6.5 Hz, 3H, CH_3) ppm. ^{13}C NMR (75.48 MHz, $CDCl_3$): δ 145.8 (C_{ipso}), 128.5 (ArCH), 127.5 (ArCH), 125.3 (ArCH), 70.4 (CH), 25.1 (CH_3) ppm.

1-(4-Methoxyphenyl)ethan-1-ol (**4b**). This product has been previously reported.[21] ^{1}H NMR (300.13 MHz, $CDCl_3$): δ 7.30 (d, *J* = 8.6 Hz, 2H, ArH), 6.88 (d, *J* = 8.6 Hz, 2H, ArH), 7.85 (q, *J* = 6.5

Hz, 1H, CH), 3.8 (s, 3H, MeO), 1.48 (d, J = 6.5 Hz, 3H, CH_3) ppm. ^{13}C NMR (75.48 MHz, $CDCl_3$): δ 159.0 (C_{ipso}), 138.0 (C_{ipso}), 130.6 (ArCH), 113.6 (ArCH), 70.0 (CH), 55.2 (OMe), 25.0 (CH_3) ppm.

1-(4-Chlorophenyl)ethan-1-ol (**4c**). This product has been previously reported.[21] ^{1}H NMR (300.13 MHz, $CDCl_3$): δ 7.30–7.25 (m, 5H, ArH), 4.88 (q, J = 6.5 Hz, 1H, CH), 1.85 (br s, 1H, OH), 1.47 (d, J = 6.5 Hz, 3H, CH_3) ppm. ^{13}C NMR (75.48 MHz, $CDCl_3$): δ 144.2 (C_{ipso}), 133.0 (C_{ipso}), 128.6 (ArCH), 126.8 (ArCH), 69.7 (CH), 25.2 (CH_3) ppm.

1-(3-Chlorophenyl)ethan-1-ol (**4d**). This product has been previously reported.[22] ^{1}H NMR (300.13 MHz, $CDCl_3$): δ 7.40–7.35 (m, 1H, ArH), 7.30–7.25 (m, 3H, ArH), 4.91 (q, J = 6.4 Hz, 1H, CH), 1.83 (br s, 1H, OH), 1.51 (d, J = 6.4 Hz, 3H, CH_3) ppm. ^{13}C NMR (75.48 MHz, $CDCl_3$): δ 147.8 (C_{ipso}), 134.4 (C_{ipso}), 129.8 (ArCH), 127.6 (ArCH), 125.6 (ArCH), 123.5 (ArCH), 69.8 (CH), 25.3 (CH_3) ppm.

1-(2-Chlorophenyl)ethan-1-ol (4e). This product has been previously reported.[22] ^{1}H NMR (300.13 MHz, $CDCl_3$): δ 7.61 (d, J = 7.7 Hz, 1H, ArH), 7.35–7.30 (m, 2H, ArH), 7.25–7.20 (m, 1H, ArH), 5.31 (q, J = 6.4 Hz, 1H, CH), 2.2 (br s, 1H, OH), 1.51 (d, J = 6.4 Hz, 3H, CH_3) ppm. ^{13}C NMR (75.48 MHz, $CDCl_3$): δ 143.0 (C_{ipso}), 131.6 (C_{ipso}), 129.4 (ArCH), 128.3 (ArCH), 127.2 (ArCH), 126.4 (ArCH), 66.9 (CH), 23.5 (CH_3) ppm.

1-(Pyridin-2-yl)ethan-1-ol (4f). This product has been previously reported.[23] ^{1}H NMR (300.13 MHz, $CDCl_3$): δ 8.55 (d, J = 4.8 Hz, 1H, ArH), 7.75–7.65 (m, 1H, ArH), 7.30 (d, J = 8.1 Hz, 1H, ArH), 7.30–7.20 (m, 1H, ArH), 4.91 (q, J = 6.5 Hz, 1H, CH), 4.35 (br s, 1H, OH), 1.52 (d, J = 6.5 Hz, 3H, CH_3) ppm. ^{13}C NMR (75.48 MHz, $CDCl_3$): δ 163.0 (C_{ipso}), 148.1 (ArCH), 136.8 (ArCH), 122.2 (ArCH), 119.8 (ArCH), 68.8 (CH), 24.25 (CH_3) ppm.

Butan-2-ol (4g). This product has been previously reported.[24] ^{1}H NMR (300.13 MHz, $CDCl_3$): δ 3.75–3.70 (m, 1H, CH), 1.67 (br s, 1H, OH), 1.50–1.45 (m, 2H, CH_2), 1.18 (d, J = 6.2 Hz, 3H, CH_3-CH), 0.93 (t, J = 7.5 Hz, 3H, CH_3-CH_2) ppm. ^{13}C NMR (75.48 MHz, $CDCl_3$): δ 69.4 (CH), 32.0 (CH_2), 22.8 (CH_3), 9.9 (CH_3) ppm.

13 Catalyst Recyclability

In a 1 mL vials equipped with a septum screw capped and a stirring bar, the catalysts **GR-MOF** (0.5-1.0 mol%) were weighed. Then, the corresponding amount of benzaldehyde (**1a**) (104 μL, 1 mmol) followed by TMSCN (136 μL, 1.1 mmol, 1.1 equiv.) were added and the reaction was stirred under inert N_2 atmosphere at room temperature overnight. After this time, 1.5 mL of DCM was added to the reaction mixture, and centrifuge at 8000 rpm for 3 min. Then, the solution was discarded, and the separated catalyst was washed with DCM (2 x 1.5 mL). Later, the catalyst was dried under vacuum and reused in a next cycle of the next reaction with the same reaction conditions previously described.

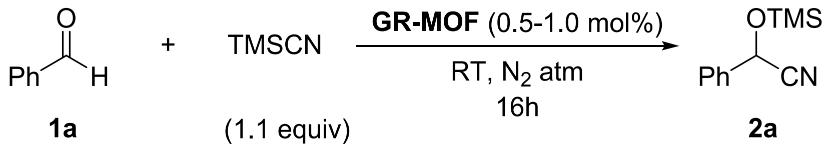

Scheme S1. Reaction conditions used for the study of recyclability of **GR-MOF** catalysts.

14 Results obtained with different lanthanides MOF catalysts

Table S8. Catalytic cyanosilylation of benzaldehyde performances of Ln-MOFs reported in the literature.

MOF	mol% MOF	mol% RE	Recyclability	Conversion	Conditions	Ratio[a]	TOF h^{-1}	Ref.
$Eu_2(MELL)(H_2O)_6$[b]	10	20	5 cycles	>99% (3h)	RT, MeCN	1:2	3.2 h^{-1}	25
[Sm/Dy/Yb(3,5-DSB)(Phen)(H_2O)]·H_2O[c]	2	2	4 cycles	>99% (3h) (Sm)<br>70%, 3h (Dy)<br>50%, 3h (Yb)	40 °C,	1:1.5	6-79 h^{-1}	26–28
Sm/Eu/Gd/Tb/Eu-Gd/Eu-Tb-psa[d]	5	5	3 cycles	70%, <2h (Eu)<br>80%, <2h (Sm)<br>87%, <2h (Eu-Gd)<br>74%, <2h (Tb)<br>94%, <2h (Eu-Tb)	RT, DCM	1:1.5	84-112 h^{-1}	29
Nd/Eu/Sm/Ho/Yb/Er-dms[e]	5	10	3 cycles	85%, <1h (Nd)<br>96%, <1h (Eu)<br>92%, <2h (Sm)<br>79%, <2h (Ho)<br>98%, <2h (Yb)	RT, DCM	1:1.5	159-234 h^{-1}	30

				93%, <2h (Er)				
Nd, Ho, Er, Yb-btc[f]	4.5	4.5	5 cycles	>99%, 2h	RT, DCM	1:2	1-11 h^{-1}	31
$Tm(BDC)_{1.5}(DMF)$-(H_2O)[g]	2	2	[h]	57%, 5h	RT	1:1	[h]	32
La/Ce/Nd/Sm/Dy$(L)(NO_3)(DMF)_2]_n \cdot n(DMF)$[i]	3	3	5 cycles	93%, 2h (La)<br>94%, 2h (Ce)<br>91%, 2h (Nd)<br>89%, 2h (Sm)<br>90%, 2h (Dy)	RT	1:4	2-16 h^{-1}	33
Tb-TCA[j]	2	2	[h]	78%, 4h	RT, DCM[k]	1:2.4	9.8 h^{-1}	34
$[Sm(L\text{-}H_2)(R\text{-}L\text{-}H_3)(H_2O)_4]\cdot nH_2O$[l]	10	10	[h]	69%, 16h	RT, DCM	1:2	0.4 h^{-1}	35
$[Yb_2(L)_2(H_2O)_3]\cdot 2H_2O$[m]	1.4	2.8	5 cycles	>99%, 24h	RT	1:2	5 h^{-1}	36
$[Pr(L^{OMe})(H_2O)_4]\cdot 2.5DMA\cdot 3H_2O$[n]	1.9	1.9	2 cycles	99%, 14h	RT	1:2	3.8 h^{-1}	37
$[Sm(H_2O)_5][Sm(H_2O)_7][Co_2Mo_{10}H_4O_{38}]\cdot 6H_2O$	2	4	3 cycles	98%, 5h	RT	1:3	9.8 h^{-1}	38
$[La/Ce/Nd(H_2O)_5]_2Mo_6V_2O_{26}\cdot 8H_2O$	1	2	3 cycles	94%, 5h (La)<br>90%, 5h (Ce)<br>96%, 5h (Nd)	RT	1:3	10.4 h^{-1}<br>10.0 h^{-1}<br>13.7 h^{-1}	39
$[Y_5L_6(OH)_3(DMF)_3]\cdot 5H_2O$[o]	0.5	2.5	7 cycles	>99%, 5h	RT	1:1	106 h^{-1}	40
$[YL(DMF)_2]\cdot(DMF)$[p]	0.5	0.5	7 cycles	>99%, 7.5h	RT	1:1	108 h^{-1}	41

Our Eu-MOF	0.5	0.5	7 cycles	>99%, 30 min	RT	1:1	1301 h^{-1}	Unpublished results

[a] Ratio between benzaldehyde **1a** and TMSCN; [b] MELL = mellitic acid; [c] 3,5-DSB = 3,5-disulfobenzoate, Phen = 1,10-phenanthroline; [d] psa = 2-phenylsuccinate; [e] dms = 2,3-dimethylsuccinate; [f] btc = 1,3,5-benzenetricarboxylate; [g] BDC = 1,4-benzenedicarboxylate; [h] Not given; [i] L = 5-[2-{2,4,6-trioxotetrahydropyrimidin-5(2*H*)-ylidene}hydrazinyl]isophthalate; [j] TCA = tricarboxytriphenylamine; [k] The aldehyde employed is 2-nitrobenzaldehyde; [l] L-H_4 = 2,2'-diethoxy-1,1'-binaphthalene-6,6'-bisphosphonic acid; [m] L = 4,4',4''-((2,4,6-trimethylbenzene-1,3,5-triyl)tris(methylene))tribenzoate; [n] L^{OMe} = 3,3'-((2,3,6,7-tetramethoxyanthracene-9,10-diyl)bis(4,1-phenylene))diacrylate.;[o] L= 3-amino-4-hydroxybenzoate; [p] {H_3L = 5-[(4-carboxyphenyl)ethynyl] isophthalic acid.

Table S9. Catalytic cyanosilylation of benzaldehyde performances of Ba, Cd, and Sr-MOFs reported in the literature.

MOF	mol% MOF	mol%	Recyclability	Conversion	Conditions	Ratio[a]	TOF h^{-1}	Ref.
$Ba_2(H_2L^{OMe})0.5(H_2O)\cdot 4H_2O$[b]	0.5	1	3 cycles	>99, 2h	RT	1:2	384	42
$[Cd_2(NiL^1)(CdL^2)][Cd_2(NiL^1)(H_2L^2)]\cdot 6DMF\cdot 5MeOH$[c]	1	5	5 cycles	93%, 63% ee, 48h	-20, DCM, PPh_3O	1:1.2	193	43
V-salen Cd-bpdc MOF[d]	0.25	0.5	3 cycles	95, 78% ee, 14h	30	1:3	27.14	44
$Cd_2(L)(DMF)_2(H_2O)_2$[e]	1	2	-	97, 1.5h	40, DCM	1:1	64.96	45
$[Cd(VOL)_2]\cdot 5H_2O$[f]	0.5	0.5	10 cycles	98, 86% ee, 24h	-78, PhMe	1:6	23.1	46
$[Cd_3(tipp)(bpdc)_2]DMA\cdot 9H_2O$[g]	0.6	1.8	5 cycles	99, 18h	RT	1:2	166.7	47
[Cd(PBA)(DMF)]DMF[h]	2	2	4 cycles	99, 8h	RT, *n*-hexane	1:2	6.25	48
$[Cd_2(1,4\text{-}NDC)_2(DMF)_2]$[i]	1.25	2.5	-	49, 72h	50, PhMe	1:1	0.54	49

[a] Ratio between benzaldehyde **1a** and TMSCN; [b] L = 5,5´-(2.3.6.7-tetramethoxyanthracene-9,10-diyl)diisophtalic acid; [c] NiL^1 = (*R,R*)-*N,N'*-bis(3-t*ert*-butyl-5-(4-pyridyl)salicylidene)-1,2-diphenyldiamine nickel(II), H_6L^2= tetra-(4-carboxyphenyl)porphyrin; [d] salen = (*R,R*)-(-)-1,2-cyclohexanediamino-*N,N'*-bis(3-*tert*-butyl-5-(4-pyridyl)salicylidene, bdpc = biphenyl-4-4'-dicarboxylic acid; [e] L = 2,3',5,5'-biphenyl tetracarboxylic acid; [f] acid-functionalized symmetrical salen ligand; [g] H_2tipp = 5,10,15,20-tetrakis(4-(imidazol-1-yl)phenyl)-porphyrin, H_2bpdc = biphenyl-4,4'-dicarboxylic acid; [h] H_2PBA = 5-(4-pyridin-3-yl-benzoylamino)-isophtalic acid; [i] (1,4-NDC) = 1,4-naphthalenedicarboxylate.

15 References

(1) Bruker AXS Inc.: Madison. Burker Apex2. B. A. I. Bruker Apex2. WI, USA 2004.

(2) Sheldrick, G. M. SADABS 1996, Program for Empirical Adsorption Correction. 1996, p Available online: https://cmacd.myweb.cs. uwindsor.

(3) Sheldrick, G. M. SHELXT - Integrated Space-Group and Crystal-Structure Determination. *Acta Crystallogr. Sect. A Found. Crystallogr.* **2015**, *71* (1), 3–8. https://doi.org/10.1107/S2053273314026370.

(4) Dolomanov, O. V.; Bourhis, L. J.; Gildea, R. J.; Howard, J. A. K.; Puschmann, H. OLEX2: A Complete Structure Solution, Refinement and Analysis Program. *J. Appl. Crystallogr.* **2009**, *42* (2), 339–341. https://doi.org/10.1107/S0021889808042726.

(5) Spek, A. L. Single-Crystal Structure Validation with the Program PLATON. *J. Appl. Crystallogr.* **2003**, *36* (1), 7–13. https://doi.org/10.1107/S0021889802022112.

(6) Frisch, M. J.; Trucks, G. W.; Schlegel, H. B.; Scuseria, G. E.; Robb, M. A.; Cheeseman, J. R.; Scalmani, G.; Barone, V.; Petersson, G. A.; Nakatsuji, H.; Li, X.; Caricato, M.; Marenich, A. V; Bloino, J.; Janesko, B. G.; Gomperts, R.; Mennucci, B.; Hratchian, H. P.; Ortiz, J. V; Izmaylov, A. F.; Sonnenberg, J. L.; Williams-Young, D.; Ding, F.; Lipparini, F.; Egidi, F.; Goings, J.; Peng, B.; Petrone, A.; Henderson, T.; Ranasinghe, D.; Zakrzewski, V. G.; Gao, J.; Rega, N.; Zheng, G.; Liang, W.; Hada, M.; Ehara, M.; Toyota, K.; Fukuda, R.; Hasegawa, J.; Ishida, M.; Nakajima, T.; Honda, Y.; Kitao, O.; Nakai, H.; Vreven, T.; Throssell, K.; Montgomery Jr., J. A.; Peralta, J. E.; Ogliaro, F.; Bearpark, M. J.; Heyd, J. J.; Brothers, E. N.; Kudin, K. N.; Staroverov, V. N.; Keith, T. A.; Kobayashi, R.; Normand, J.; Raghavachari, K.; Rendell, A. P.; Burant, J. C.; Iyengar, S. S.; Tomasi, J.; Cossi, M.; Millam, J. M.; Klene, M.; Adamo, C.; Cammi, R.; Ochterski, J. W.; Martin, R. L.; Morokuma, K.; Farkas, O.; Foresman, J. B.; Fox, D. J. Gaussian16 {R}evision {C}.01. 2016.

(7) Becke, A. D. Density-functional Thermochemistry. III. The Role of Exact Exchange. *J. Chem. Phys.* **1993**, *98* (7), 5648–5652. https://doi.org/10.1063/1.464913.

(8) Lee, C.; Yang, W.; Parr, R. G. Development of the Colle-Salvetti Correlation-Energy Formula into a Functional of the Electron Density. *Phys. Rev. B* **1988**, *37* (2), 785–789. https://doi.org/10.1103/PhysRevB.37.785.

(9) Ditchfield, R.; Hehre, W. J.; Pople, J. A. Self-Consistent Molecular-Orbital Methods. IX. An Extended Gaussian-Type Basis for Molecular-Orbital Studies of Organic Molecules. *J. Chem. Phys.* **1971**, *54* (2), 720–723. https://doi.org/10.1063/1.1674902.

(10) O'boyle, N. M.; Tenderholt, A. L.; Langner, K. M. Cclib: A Library for Package-Independent Computational Chemistry Algorithms. *J. Comput. Chem.* **2008**, *29* (5), 839–845. https://doi.org/https://doi.org/10.1002/jcc.20823.

(11) Dennington, R.; Keith, T. A.; Millam, J. M. GaussView {V}ersion {6}. 2019.

(12) Thavornpradit, S.; Killough, J. M.; Bergbreiter, D. E. Minimizing Solvent Waste in Catalytic Reactions in Highly Recyclable Hydrocarbon Solvents. *Org. Biomol. Chem.* **2020**, *18* (22), 4248–4256. https://doi.org/10.1039/d0ob00734j.

(13) Wang, W.; Luo, M.; Yao, W.; Ma, M.; Pullarkat, S. A.; Xu, L.; Leung, P. H. Catalyst-Free and Solvent-Free Cyanosilylation and Knoevenagel Condensation of Aldehydes. *ACS Sustain. Chem. Eng.* **2019**, *7* (1), 1718–1722. https://doi.org/10.1021/acssuschemeng.8b05486.

(14) Gu, J. Z.; Wan, S. M.; Kirillova, M. V.; Kirillov, A. M. H-Bonded and Metal(Ii)-Organic Architectures Assembled from an Unexplored Aromatic Tricarboxylic Acid: Structural Variety and Functional Properties. *Dalt. Trans.* **2020**, *49* (21), 7197–7209. https://doi.org/10.1039/d0dt01261k.

(15) Wu, W. B.; Zeng, X. P.; Zhou, J. Carbonyl-Stabilized Phosphorus Ylide as an Organocatalyst for Cyanosilylation Reactions Using TMSCN. *ACS Appl. Mater. Interfaces* **2020**, *85*. https://doi.org/10.1021/acs.joc.9b03347.

(16) Sharma, M. K.; Singh, D.; Mahawar, P.; Yadav, R.; Nagendran, S. Catalytic Cyanosilylation Using Germylene Stabilized Platinum(Ii) Dicyanide. *Dalt. Trans.* **2018**, *47* (17), 5943–5947. https://doi.org/10.1039/c8dt00043c.

(17) Harinath, A.; Bhattacharjee, J.; Nayek, H. P.; Panda, T. K. Alkali Metal Complexes as Efficient Catalysts for Hydroboration and Cyanosilylation of Carbonyl Compounds. *Dalt. Trans.* **2018**, *47* (36), 12613–12622. https://doi.org/10.1039/c8dt02032a.

(18) Garnes-Portolés, F.; Rivero-Crespo, M. Á.; Leyva-Pérez, A. Nanoceria as a Recyclable Catalyst/Support for the Cyanosilylation of Ketones and Alcohol Oxidation in Cascade. *J. Catal.* **2020**, *392*, 21–28. https://doi.org/10.1016/j.jcat.2020.09.032.

(19) Nie, Y. M.; Li, S. H.; Lin, M. Y.; Yan, J. A Micro-Environment Tuning Approach for Enhancing the Catalytic Capabilities of Lanthanide Containing Polyoxometalate in the Cyanosilylation of Ketones. *Chem. Commun.* **2020**, *56* (26), 3809–3812. https://doi.org/10.1039/d0cc01216e.

(20) Sternberg, Jeffrey Arthur; Adams, J. B. EP0503798A1_Original_document_20210421152633.Pdf. 1992, p 120.

(21) Yadav, S.; Vijayan, P.; Yadav, S.; Gupta, R. Ruthenium Complexes of Phosphine-Amide Based Ligands as Efficient Catalysts for Transfer Hydrogenation Reactions. *Dalt. Trans.* **2021**, *50* (9), 3269–3279. https://doi.org/10.1039/d0dt04401f.

(22) Luo, N.; Zhong, Y.; Liu, J. T.; Ouyang, L.; Luo, R. An Efficient Hydration and Tandem Transfer Hydrogenation of Alkynes for the Synthesis of Alcohol in Water. *Synth.* **2020**, *52* (22), 3439–3445. https://doi.org/10.1055/s-0040-1707233.

(23) Bhattacharya, P.; Krause, J. A.; Guan, H. Iron Hydride Complexes Bearing Phosphinite-Based Pincer Ligands: Synthesis, Reactivity, and Catalytic Application in Hydrosilylation Reactions. *Organometallics* **2011**, *30* (17), 4720–4729. https://doi.org/10.1021/om2005589.

(24) Clarke, Z. E.; Maragh, P. T.; Dasgupta, T. P.; Gusev, D. G.; Lough, A. J.; Abdur-Rashid, K. A Family of Active Iridium Catalysts for Transfer Hydrogenation of Ketones. *Organometallics* **2006**, *25* (17), 4113–4117. https://doi.org/10.1021/om060049z.

(25) Batista, P. K.; Alves, D. J. M.; Rodrigues, M. O.; De Sá, G. F.; Junior, S. A.; Vale, J. A. Tuning the Catalytic Activity of Lanthanide-Organic Framework for the Cyanosilylation of Aldehydes. *J. Mol. Catal. A Chem.* **2013**, *379*, 68–71. https://doi.org/10.1016/j.molcata.2013.07.016.

(26) Dvries, R. F.; De La Peña-Oshea, V. A.; Snejko, N.; Iglesias, M.; Gutiérrez-Puebla, E.; Monge, M. Á. Insight into the Correlation between Net Topology and Ligand Coordination Mode in New Lanthanide MOFs Heterogeneous Catalysts: A Theoretical and Experimental Approach. *Cryst. Growth Des.* **2012**, *12* (11), 5535–5545. https://doi.org/10.1021/cg301096d.

(27) Dvries, R. F.; Iglesias, M.; Snejko, N.; Gutiérrez-Puebla, E.; Monge, M. A. Lanthanide Metal-Organic Frameworks: Searching for Efficient Solvent-Free Catalysts. *Inorg. Chem.* **2012**, *51* (21), 11349–11355. https://doi.org/10.1021/ic300816r.

(28) Dvries, R. F.; Snejko, N.; Iglesias, M.; Gutiérrez-Puebla, E.; Monge, M. A. Ln-MOF Pseudo-Merohedral Twinned Crystalline Family as Solvent-Free Heterogeneous Catalysts. *Cryst. Growth Des.* **2014**, *14* (5), 2516–2521. https://doi.org/10.1021/cg5002336.

(29) Gomez, G. E.; Kaczmarek, A. M.; Van Deun, R.; Brusau, E. V.; Narda, G. E.; Vega, D.; Iglesias, M.; Gutierrez-Puebla, E.; Monge, M. Á. Photoluminescence, Unconventional-Range Temperature Sensing, and Efficient Catalytic Activities of Lanthanide Metal-Organic Frameworks. *Eur. J. Inorg. Chem.* **2016**, *2016* (10), 1577–1588. https://doi.org/10.1002/ejic.201501402.

(30) Gomez, G. E.; Brusau, E. V.; Sacanell, J.; Soler Illia, G. J. A. A.; Narda, G. E. Insight into the Metal Content-Structure-Property Relationship in Lanthanide Metal-Organic Frameworks: Optical Studies, Magnetism, and Catalytic Performance. *Eur. J. Inorg. Chem.* **2018**, *2018* (20–21), 2452–2460. https://doi.org/10.1002/ejic.201701474.

(31) Gustafsson, M.; Bartoszewicz, A.; Martiín-Matute, B.; Sun, J.; Grins, J.; Zhao, T.; Li, Z.; Zhu, G.; Zou, X. A Family of Highly Stable Lanthanide Metal-Organic Frameworks: Structural Evolution and Catalytic Activity. *Chem. Mater.* **2010**, *22* (11), 3316–3322. https://doi.org/10.1021/cm100503q.

(32) He, H.; Ma, H.; Sun, D.; Zhang, L.; Wang, R.; Sun, D. Porous Lanthanide-Organic Frameworks: Control over Interpenetration, Gas Adsorption, and Catalyst Properties. *Cryst. Growth Des.* **2013**, *13* (7), 3154–3161. https://doi.org/10.1021/cg400531j.

(33) Karmakar, A.; Rúbio, G. M. D. M.; Paul, A.; Guedes da Silva, M. F. C.; Mahmudov, K. T.; Guseinov, F. I.; Carabineiro, S. A. C.; Pombeiro, A. J. L. Lanthanide Metal Organic Frameworks Based on Dicarboxyl-Functionalized Arylhydrazone of Barbituric Acid: Syntheses, Structures, Luminescence and Catalytic Cyanosilylation of Aldehydes. *Dalt. Trans.* **2017**, *46* (26), 8649–8657. https://doi.org/10.1039/c7dt01056g.

(34) Wu, P.; Wang, J.; Li, Y.; He, C.; Xie, Z.; Duan, C. Luminescent Sensing and Catalytic Performances of a Multifunctional Lanthanide-Organic Framework Comprising a Triphenylamine Moiety. *Adv. Funct. Mater.* **2011**, *21* (14), 2788–2794. https://doi.org/10.1002/adfm.201100115.

(35) Evans, O. R.; Ngo, H. L.; Lin, W. Chiral Porous Solids Based on Lamellar Lanthanide Phosphonates [5]. *J. Am. Chem. Soc.* **2001**, *123* (42), 10395–10396.

https://doi.org/10.1021/ja0163772.

(36) Wang, X.; Zhang, L.; Yang, J.; Liu, F.; Dai, F.; Wang, R.; Sun, D. Lanthanide Metal-Organic Frameworks Containing a Novel Flexible Ligand for Luminescence Sensing of Small Organic Molecules and Selective Adsorption. *J. Mater. Chem. A* **2015**, *3* (24), 12777–12785. https://doi.org/10.1039/c5ta00061k.

(37) Liu, X.; Lin, H.; Xiao, Z.; Fan, W.; Huang, A.; Wang, R.; Zhang, L.; Sun, D. Multifunctional Lanthanide–Organic Frameworks for Fluorescent Sensing, Gas Separation and Catalysis. **2016**, *45*, 3743–3749. https://doi.org/10.1039/c5dt04339e.

(38) An, H.; Wang, L.; Hu, Y.; Fei, F. Temperature-Induced Racemic Compounds and Chiral Conglomerates Based on Polyoxometalates and Lanthanides: Syntheses, Structures and Catalytic Properties. *CrystEngComm* **2015**, *17* (7), 1531–1540. https://doi.org/10.1039/c4ce01802h.

(39) Fei, F.; An, H.; Meng, C.; Wang, L.; Wang, H. Lanthanide-Supported Molybdenum-Vanadium Oxide Clusters: Syntheses, Structures and Catalytic Properties. *RSC Adv.* **2015**, *5* (24), 18796–18805. https://doi.org/10.1039/c4ra16237d.

(40) Echenique-Errandonea, E.; Pérez, J. M.; Rojas, S.; Cepeda, J.; Seco, J. M.; Fernández, I.; Rodríguez-Diéguez, A. A Novel Yttrium-Based Metal-Organic Framework for the Efficient Solvent-Free Catalytic Synthesis of Cyanohydrin Silyl Ethers. *Dalt. Trans.* **2021**, *50* (34), 11720–11724. https://doi.org/10.1039/d1dt01953h.

(41) Pérez, J. M.; Rojas, S.; García-García, A.; Montes-Andrés, H.; Ruiz Martínez, C.; Romero-Cano, M. S.; Choquesillo-Lazarte, D.; Abdelkader-Fernández, V. K.; Pérez-Mendoza, M.; Cepeda, J.; Rodríguez-Diéguez, A.; Fernández, I. Catalytic Performance and Electrophoretic Behavior of an Yttrium-Organic Framework Based on a Tricarboxylic Asymmetric Alkyne. *Inorg. Chem.* **2022**, *61* (3), 1377–1384. https://doi.org/10.1021/acs.inorgchem.1c02864.

(42) Liu, F.; Xu, Y.; Zhao, L.; Zhang, L.; Guo, W.; Wang, R.; Sun, D. Porous Barium-Organic Frameworks with Highly Efficient Catalytic Capacity and Fluorescence Sensing Ability. *J. Mater. Chem. A* **2015**, *3* (43), 21545–21552. https://doi.org/10.1039/c5ta03680a.

(43) Li, J.; Ren, Y.; Qi, C.; Jiang, H. The First Porphyrin-Salen Based Chiral Metal-Organic Framework for Asymmetric Cyanosilylation of Aldehydes. *Chem. Commun.* **2017**, *53* (58), 8223–8226. https://doi.org/10.1039/c7cc03499g.

(44) Bhunia, A.; Dey, S.; Moreno, J. M.; Diaz, U.; Concepcion, P.; Van Hecke, K.; Janiak, C.; Van Der Voort, P. A Homochiral Vanadium-Salen Based Cadmium Bpdc MOF with Permanent Porosity as an Asymmetric Catalyst in Solvent-Free Cyanosilylation. *Chem. Commun.* **2016**, *52* (7), 1401–1404. https://doi.org/10.1039/c5cc09459c.

(45) Zhang, X. N.; Liu, L.; Han, Z. B.; Gao, M. L.; Yuan, D. Q. A Dual-Functional Cd(II)-Organic-Framework Demonstrating Selective Sensing of Zn2+ and Fe3+ Ions Exclusively and Size-Selective Catalysis towards Cyanosilylation. *RSC Adv.* **2015**, *5* (14), 10119–10124. https://doi.org/10.1039/c4ra11678j.

(46) Zhu, C.; Xia, Q.; Chen, X.; Liu, Y.; Du, X.; Cui, Y. Chiral Metal−Organic Framework as a Platform for Cooperative Catalysis in Asymmetric Cyanosilylation of Aldehydes. *ACS Catal.* **2016**, *6* (11), 7590–7596. https://doi.org/10.1021/acscatal.6b02359.

(47) Jiang, W.; Yang, J.; Liu, Y. Y.; Song, S. Y.; Ma, J. F. A Stable Porphyrin-Based Porous Mog Metal-Organic Framework as an Efficient Solvent-Free Catalyst for C-C Bond Formation. *Inorg. Chem.* **2017**, *56* (5), 3036–3043. https://doi.org/10.1021/acs.inorgchem.6b03174.

(48) Hu, L.; Hao, G. X.; Luo, H. D.; Ke, C. X.; Shi, G.; Lin, J.; Lin, X. M.; Qazi, U. Y.; Cai, Y. P. Bifunctional 2D Cd(II)-Based Metal-Organic Framework as Efficient Heterogeneous Catalyst for the Formation of C-C Bond. *Cryst. Growth Des.* **2018**, *18* (5), 2883–2889. https://doi.org/10.1021/acs.cgd.7b01728.

(49) Choi, I. H.; Kim, Y.; Lee, D. N.; Huh, S. Three-Dimensional Cobalt(II) and Cadmium(II) MOFs Containing 1,4-Naphthalenedicarboxylate: Catalytic Activity of Cd-MOF. *Polyhedron* **2016**, *105*, 96–103. https://doi.org/10.1016/j.poly.2015.12.022.
